# Effectiveness of comprehensive geriatric assessment intervention on quality of life, caregiver burden and length of hospital stay: a systematic review and meta-analysis of randomised controlled trials

**DOI:** 10.1186/s12877-021-02319-2

**Published:** 2021-06-21

**Authors:** Zhongyi Chen, Zhaosheng Ding, Caixia Chen, Yangfan Sun, Yuyu Jiang, Fenglan Liu, Shanshan Wang

**Affiliations:** 1 Research Office of Chronic Disease Management and Rehabilitation, Wuxi School of Medicine, Jiangnan University, No. 1800 Lihu Avenue, Wuxi, Jiangsu Province China; 2 Rongjun Hospital of Jiangsu Province, Wuxi, China; 3 Wuxi Tongren Rehabilitation Hospital, Wuxi, China; 4 Medical School, Liaocheng University, Liaocheng, China

**Keywords:** Comprehensive geriatric assessment intervention, Quality of life, Caregiver burden, Length of hospital stay, Meta-analysis

## Abstract

**Background:**

Comprehensive geriatric assessment (CGA) interventions can improve functional ability and reduce mortality in older adults, but the effectiveness of CGA intervention on the quality of life, caregiver burden, and length of hospital stay remains unclear. The study aimed to determine the effectiveness of CGA intervention on the quality of life, length of hospital stay, and caregiver burden in older adults by conducting meta-analyses of randomised controlled trials (RCTs).

**Methods:**

A literature search in PubMed, Embase, and Cochrane Library was conducted for papers published before February 29, 2020, based on inclusion criteria. Standardised mean difference (SMD) or mean difference (MD) with 95% confidence intervals (CIs) was calculated using the random-effects model. Subgroup analyses, sensitivity analyses, and publication bias analyses were also conducted.

**Results:**

A total of 28 RCTs were included. Overall, the intervention components common in different CGA intervention models were interdisciplinary assessments and team meetings. Meta-analyses showed that CGA interventions improved the quality of life of older people (SMD = 0.12; 95% CI = 0.03 to 0.21; *P* = 0.009) compared to usual care, and subgroup analyses showed that CGA interventions improved the quality of life only in participants’ age > 80 years and at follow-up ≤3 months. The change value of quality of life in the CGA intervention group was better than that in the usual care group on six dimensions of the 36-Item Short-Form Health Survey questionnaire (SF-36). Also, compared to usual care, the CGA intervention reduced the caregiver burden (SMD = − 0.56; 95% CI = − 0.97 to − 0.15, *P* = 0.007), but had no significant effect on the length of hospital stay.

**Conclusions:**

CGA intervention was effective in improving the quality of life and reducing caregiver burden, but did not affect the length of hospital stay. It is recommended that future studies apply the SF-36 to evaluate the impact of CGA interventions on the quality of life and provide supportive strategies for caregivers as an essential part of the CGA intervention, to find additional benefits of CGA interventions.

**Supplementary Information:**

The online version contains supplementary material available at 10.1186/s12877-021-02319-2.

## Background

People aged > 60 years will account for 22% of the world’s population by 2050 [1]. With worldwide population aging, increasing numbers of older adults are living with multiple chronic conditions and complex psychological and social problems [2]. Comprehensive Geriatric Assessment (CGA) is a multidisciplinary diagnostic and therapeutic intervention process centred on the older adults, through a comprehensive assessment of the physical, psychological, functional and socio-economic dimensions, to develop a comprehensive individual treatment plan for the betterment of the overall health of the older adults [3]. As a core technology in geriatrics, CGA has developed different intervention models applied in hospitals, outpatient clinics and in the community [4]. Numerous reviews have confirmed the benefits of CGA interventions for older adults compared to usual care: improved functional ability [4–6], reduced mortality [3, 4, 7], more probability to live at home after discharge [5, 8]. However, the effects of CGA interventions on the quality of life, caregiver burden and length of hospital stay are not yet clear [9, 10].

Quality of life is an important outcome measure in clinical research and its multidimensional connotations are aligned with the multidimensional intervention characteristics of CGA [11], enabling a comprehensive reflection of the effects of CGA interventions from the patient-reported perspective [12]. There were only 2 systematic reviews evaluating the impact of CGA interventions on the quality of life of older adults [13, 14]. One of the reviews only included 1 clinical trial and was not able to conduct meta-analysis, with the 36-Item Short Form Health Survey questionnaire (SF-36) as the evaluation instrument, and the results suggested that the CGA intervention group scored higher than the usual care group on physical component summary and mental component summary, which were statistically different but did not meet the criteria for clinical improvement [13]. Another review showed that neither of the two CGA intervention models (CGA unit and CGA-team) had an effect on the quality of life compared to usual care, and proposed that strength of the evidence for the finding was limited and that there was a need to further explore the impact of CGA interventions on quality of life [14]. The World Health Organization (WHO) guidelines on “Integrated Caring for Older People” pointed out that caregiver burden should be a concern when caring for older people [15]. CGA interventions are primarily targeted at older people. There were only 2 systematic reviews that attempted to evaluate the impact of CGA interventions on the caregiver burden, but both did not report the results due to the lack of relevant studies [13, 14]. The length of hospital stay is an important economic outcome measure for evaluating CGA interventions. Some previous reviews only summarised the effects of CGA interventions on the length of hospital stay descriptively and did not conduct meta-analyses to clarify the specific effects [5, 6, 8, 16]. Other reviews conducted meta-analyses, but their results were controversial [17–20].

Previous systematic reviews and meta-analyses related to CGA evaluated the effectiveness of one or two CGA intervention models [5–8, 13, 14, 16–20], with only Stuck et al. [4] in 1993 providing a comprehensive evaluation of the effectiveness of various CGA intervention models. Therefore, this study comprehensively collected randomised controlled trials (RCTs) of various CGA intervention models and conducted meta-analyses to determine the effectiveness of CGA interventions on the quality of life, caregiver burden, and length of hospital stay for older adults.

## Methods

This systematic review was reported according to the recommendations in the Preferred Reporting Items for Systematic Reviews and Meta-Analyses (PRISMA) main statement [21]. PRISMA checklist can be found in Additional file 1. It was registered at the International Prospective Register of Systematic Reviews database (PROSPERO: CRD42020178811).

### Search strategy

The electronic databases of PubMed, Embase and Cochrane Library were systematically searched for studies published before February 29, 2020, with language restriction to English. The search strategy was developed initially in PubMed and then adapted for other databases. The search strategies of the three databases are available in Additional file 2. The reference lists of retrieved studies and previous relevant reviews were reviewed by manual searches to select any additional eligible studies.

### Study selection and exclusion criteria

Two researchers (C.Z.Y. and D.Z.S.) independently screened all titles and abstracts from the search results. Full-text articles for all potentially eligible studies were independently reviewed by each researcher to determine final inclusion. Disagreements between the two researchers were resolved through discussion, with the third researcher (C.C.X.) available to address any disagreements.

Studies were eligible for inclusion if they met the following criteria: (1) participants: the age of adults meets the criteria for older people in the country where the study was being conducted. (2) intervention: various comprehensive geriatric assessment interventions were included. They are divided into inpatient CGA and outpatient CGA [4, 9]. The inpatient CGA includes two types of intervention models [4, 9]. The first is the CGA-team, which is delivered in the non-geriatric ward by a mobile multidisciplinary team as a consultation [4, 9]. The second is the CGA-unit. It is provided in geriatric-based wards or units by a multidisciplinary team that is the primary care provider for the older adults [4, 9]. The outpatient CGA includes three types of intervention models [4, 9]. The first is the outpatient assessment service (OAS) with CGA intervention provided in an outpatient setting [4, 9]. The second is the hospital home assessment service (HHAS) with in-home CGA intervention for patients recently discharged from the hospital [4, 9]. The third is home assessment service (HAS) with in-home CGA intervention for community-dwelling older people [4, 9]. (3) comparator: control group with usual care was included. (4) outcomes: studies that reported any of the following outcome measures were included: quality of life, caregiver burden and length of hospital stay. (5) study design: only RCTs were included. (6) publication type: only full original publications were considered. (7) language: only studies published in English were included.

Studies without the data of result were excluded. When there were duplicate reports in different studies, only the study providing the most detailed data were included in this systematic review. Stepped wedge cluster randomised controlled trials were also excluded.

### Data extraction

Data were extracted from the included studies by two independent researchers (C.Z.Y. and D.Z.S.) respectively. The details of extracted data were shown in Table 1 and Additional file 3. Disagreements in data extraction between the two researchers were resolved through discussion to gain agreement, with the third researcher (C.C.X) available to address any disagreements.

**Table 1:** Summary of outcomes of included studies

Study	Outcomes			Evaluation instruments
	Quality of life (measure time)	Caregiver burden (measure time)	Length of hospital stay (measure time)	
Gayton 1987 [28]			√ (Discharge)	
Hogan 1987 [29]			√ (Discharge)	
Thomas 1993 [30]			√ (Discharge)	
Nikolaus 1999 (CGA) [31]			√ (Discharge; at follow up 12 months)	
Nikolaus 1999 (CGA + home) [31]			√ (Discharge; at follow up 12 months)	
Naglie 2002 [32]			√ (Discharge; at follow up 6 months ǂ)	
Shyu 2005 [33]	√ (1 month after discharge ǂ; 3 months after discharge ǂ)		√ (Discharge)	Quality of life: the 36-Item Short Form Health Survey questionnaire(SF-36) ǁ
Vidan 2005 [34]			√ (Discharge)	
Kircher 2007 [35]	√ (At follow up 3 months; at follow up 12 months)		√ (At follow up 12 months)	Quality of life: Philadelphia Geriatric Centre Morale Scale(PGCMS)
Pitkala 2008 [36]	√ (Discharge)			Quality of life: 15D questionnaire
Prestmo 2015 [37]	√ (1 month after surgery; 4 month after surgery; 12 month after surgery)		√ (Discharge)	Quality of life:EQ-5D-3L
Partridge 2017* [38]			√ (Discharge ǂ)	
Applegate 1990* [39]			√ (12 months after randomisation ǂ)	
Karppi 1995 [40]			√ (At follow up 12 months; at follow up 24 months)	
Covinsky 1997 [41]		√ (90 days after dischargeǂ)	√ (Discharge)	Caregiver burden: The Robinson Caregiver Strain Index ǁ
Asplund 2000 [42]			√ (Discharge)	
Counsell 2000 [43]			√ (Discharge)	
Cohen 2002(unit) [44]	√ (Discharge; 12 months after randomisation)			Quality of life: the 36-Item Short Form Health Survey questionnaire(SF-36)
Saltvedt 2004 [45]			√ (Discharge; at follow up 6 months)	
Ekerstad 2016 [46]	√ (Discharge; at follow up 3 months)		√ (Discharge; at follow up 3 months)	Quality of life: EuroQoL-visual analog scale (EQ-VAS); Health Utilities Index-3 ǁ
Silverman 1995 [47]		√ (12 months after randomisation)		Caregiver burden: Family strain scale developed and validated by Morycz; a global burden questionnaire ǁ
Reuben 1999 [48]	√ (At follow up 15 months)			Quality of life: the 36-Item Short Form Health Survey questionnaire(SF-36)
Burns 2000 [49]	√ (At follow up 12 months; at follow up 24 months)			Quality of life: Rand general well-being (GWB) inventory; Center for Epidemiologic Studies-Depression (CES-D) scale ǁ; perceived global life satisfaction (GLS) ǁ
Weuve 2000 [50]		√ (At follow up 12 months)		Caregiver burden: previously developed inventory that consists of 22 equally weighted statements about perception of burden
Cohen 2002(outpatient) [44]	√ (12 months after randomisation)			Quality of life: the 36-Item Short Form Health Survey questionnaire(SF-36)
Ekdahl 2015 [51]	√ (At follow up 12 months; at follow up 24 months)		√ (At follow up 24 months)	Quality of life: EQ-5D-3L
Zintchouk 2018* [52]	√ (At follow up 3 months ǂ) number of improvement		√ (At follow up 3 months ǂ)	Quality of life: Depression List (DL) ǁ
Counsell 2007 [53]	√ (At follow up 24 months)		√ (At follow up 12 months ǂ; at follow up 24 months ǂ)	Quality of life: the 36-Item Short Form Health Survey questionnaire(SF-36)
Fairhall 2015 [54]	√ (At follow up 3 months; at follow up 12 months)			Quality of life: EQ-5D
Edmans 2013 [55]	√ (At follow up 90 days)			Quality of life: EQ-5D; ICEpop CA Pability measure for older people (ICECAP-O) ǁ

CGA, comprehensive geriatric assessment

^*^ study not included in meta-analysis; ǂoutcome data at the endpoint not included in meta-analysis; ǁ outcome data measured by the evaluation instruments not included in meta-analysis

### Assessment of risk of bias

Two researchers (C.Z.Y. and D.Z.S.) independently assessed the risk of bias of the included RCTs using the revised Cochrane risk-of-bias tool for randomised trials (RoB2) [22]. Risk of bias was evaluated in the five different domains: (1) randomisation process; (2) deviations from intended interventions; (3) missing outcome data; (4) measurement of the outcome; and (5) bias in selection of the reported result. Each domain was rated as having a low risk of bias, some concerns, or a high risk of bias. Because the risk of bias assessed is for a specific outcome or endpoint [22], we evaluated the risk of bias for each study separately for different evaluation indicators and for different outcome measure time. If any disagreements existed, the third researcher (J.Y.Y) was consulted to reach an agreement.

### Data synthesis and analysis

Results were quantitatively synthetized by means of meta-analysis using Review Manager (RevMan) version 5.3 software. For studies that reported only means, standard errors, t values or *P* values, we calculated standard deviations (SD) [23–26]. For studies that reported only range or 95% confidence intervals (CIs), we calculated means [23–26]. We also calculated means and SD of change values based on specific data at baseline and at endpoint [26]. Where possible and available, intent-to-treat data were preferred. For the continuous outcomes “quality of life” and “caregiver burden”, standardised mean difference (SMD) and 95% CIs were reported. The SMD is used as a summary statistic in meta-analysis when studies assess the same outcome but measure it in different ways [26]. For the continuous outcomes “length of hospital stay”, mean difference (MD) and 95% CIs were presented. A priori random-effects model was preferred based on the foreseeable complexity and multicomponent nature of CGA interventions. For each meta-analysis, statistical heterogeneity was assessed using the Cochran Q test and *I*^*2*^ statistic [26, 27]. If a *P* value < 0.1 or an *I*^*2*^ value > 50%, it represents a substantial or considerable heterogeneity [26]. If the pooled result included clinical heterogeneity, subgroup analysis was performed to search for the source of heterogeneity. Subgroup analysis was performed based on intervention model, participants’ age, outcome measure time and evaluation instruments. Publication bias was explored by constructing the funnel plot and computing the Egger test using metafunnel and metabias commands in Stata software version 12.0 respectively. A leave-one-out sensitivity analysis and sensitivity analyses based on risk of bias were performed to check the robustness of the results.

## Results

### Selection of studies

**Fig. 1:**
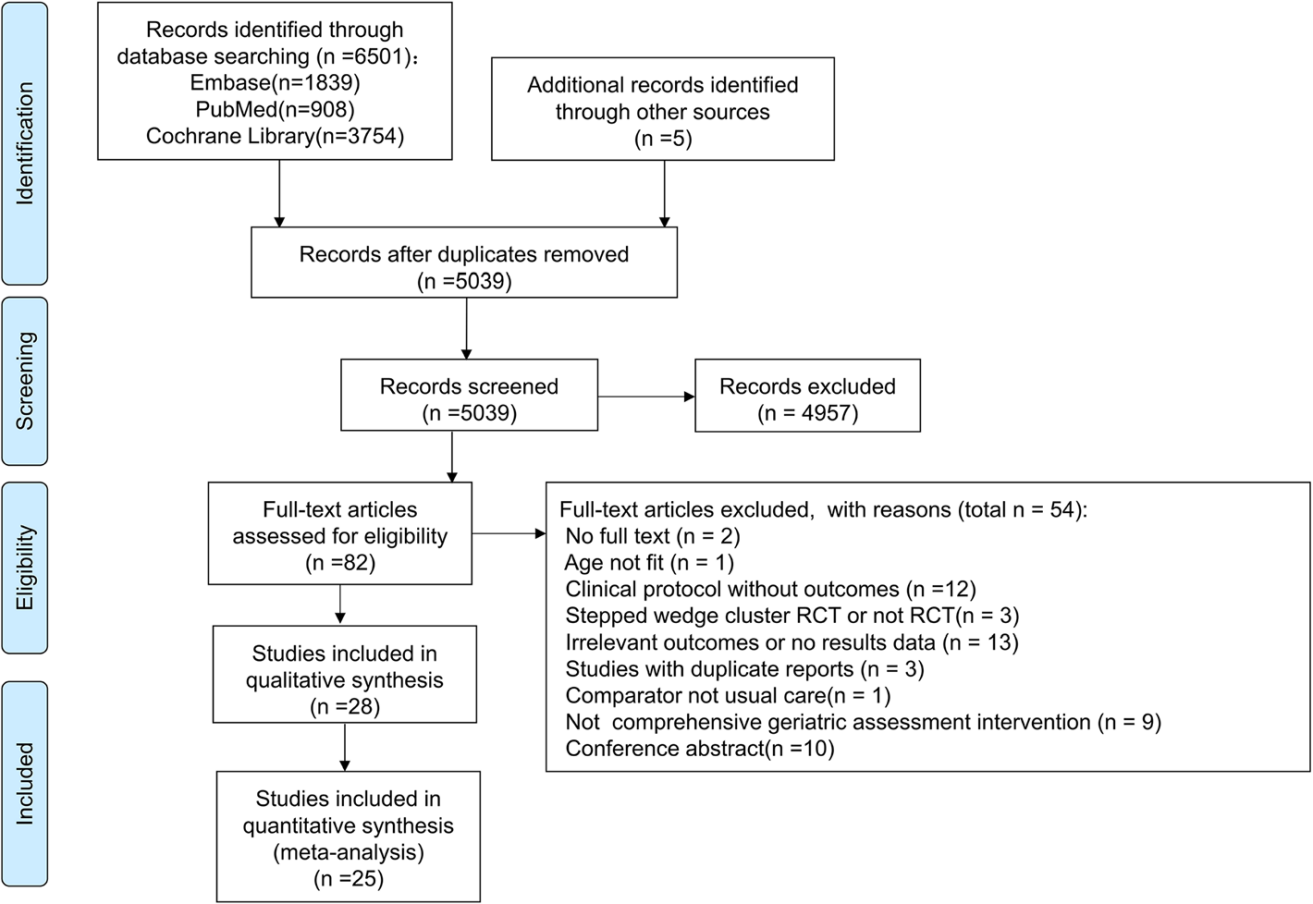
PRISMA flowchart

Figure 1 showed the search and selection of studies based on the PRISMA flowcharts. The electronic search retrieved a total of 6501 relevant studies in the three databases, and 5 additional studies were identified by hand searching of these studies. Of these studies, 1467 studies were removed due to duplicates, and 4957 studies were excluded based on title and abstract content. The remaining 82 studies were retrieved for further full-text evaluations, 54 of which were excluded because they did not meet eligibility criteria. Finally, 28 randomised clinical trials were included in this review [28–55], and 25 randomised clinical trials were included in the meta-analysis [28–37, 40–51, 53–55].

### Characteristics of included studies

We counted Cohen 2002 [44] as two studies (Cohen 2002(unit); Cohen 2002(outpatient)), as the researches used a 2 × 2 factorial design that compared care received in an inpatient geriatric evaluation and management unit versus usual care, followed by outpatient geriatric evaluation and management versus usual outpatient care. We also counted Nikolaus 1999 [31] as two studies (Nikolaus 1999(CGA); Nikolaus 1999(CGA + home)) owing to the different CGA interventions evaluated. Therefore, a total of 13,261 participants from 30 studies were included in this study. A summary of the 30 included studies is presented in Table 1 and Additional file 3. Studies were published between 1987 and 2018. The CGA intervention model included in the study consisted of CGA-team (*n* = 12) [28–38], CGA-unit (*n* = 8) [39–46], OAS (*n* = 7) [44, 47–52], HHAS (*n* = 2) [53, 54] and HAS (n = 1) [55]. The sample size of the studies varied from 98 to 1388 subjects. The mean age of participants ranged from 71.8 years to 85.7 years. The intervention components common in different CGA intervention models were interdisciplinary assessments (*n* = 30) [28–55] and team meetings (*n* = 15) [28–30, 32, 34, 35, 39, 42–45, 48, 51, 53, 54]. Fourteen studies used quality of life as an outcome measure [33, 35–37, 44, 46, 48, 49, 51–55], and 12 studies could be included in a meta-analysis [35–37, 44, 46, 48, 49, 51, 53–55]. The outcome measure time varied ranging from discharge to follow-up for 24 months. Three studies [41, 47, 50] used caregiver stress as an evaluation indicator and 2 studies [47, 50] could be included in a meta-analysis. The outcome measure time ranged from the 90 day after discharge to follow-up for 12 months. Twenty-one [28–35, 37–43, 45, 46, 51–53] studies used length of hospital stay as an evaluation indicator and 17 studies [28–31, 33–35, 37, 40–43, 45, 46, 51] could be included in a meta-analysis. The outcome measure time ranged from discharge to follow-up for 24 months.

### Risk of bias

**Fig. 2:**
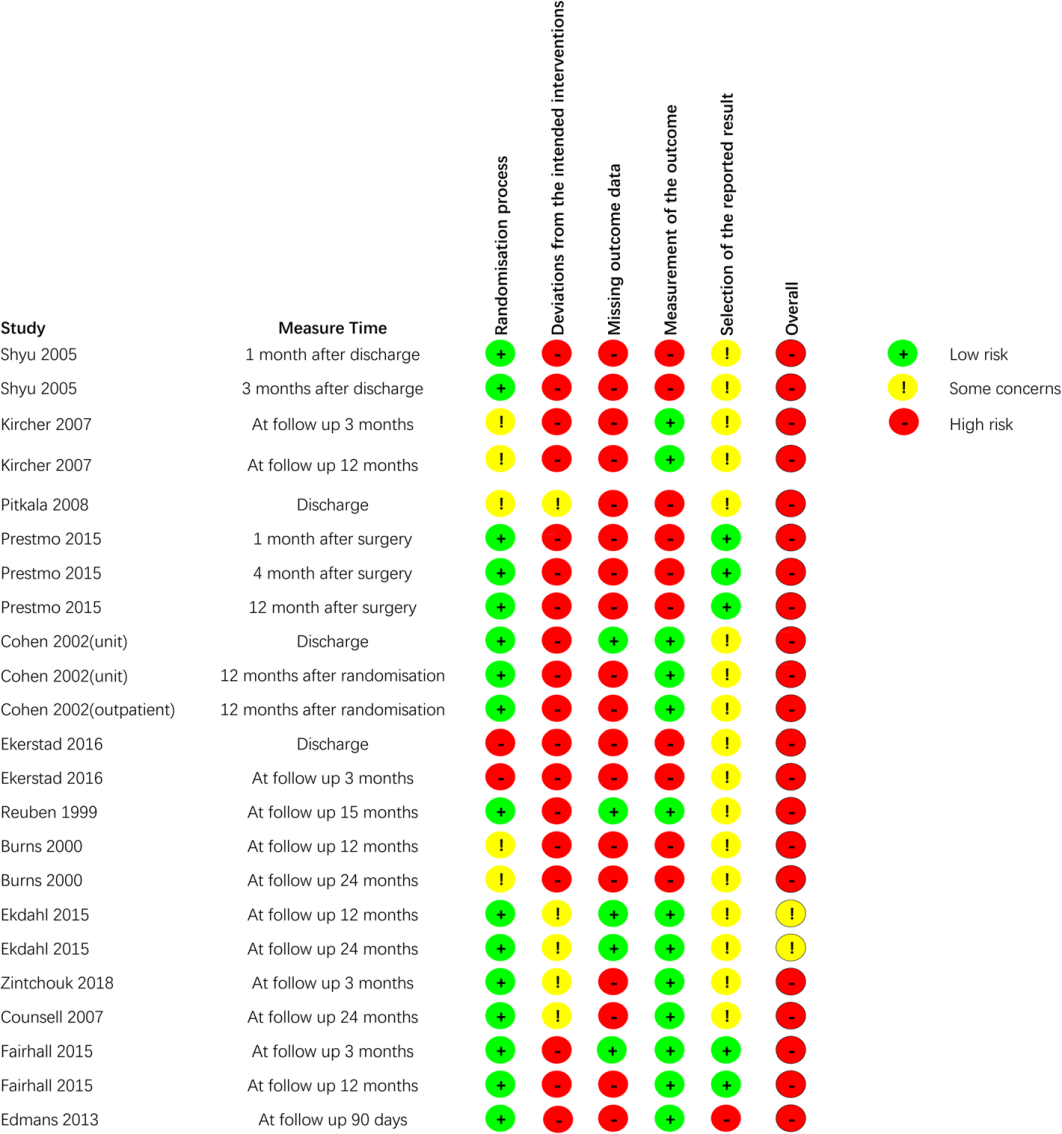
Risk of bias of included studies about the outcome indicator quality of life

Assessments of the risk of bias are shown in Fig. 2, Additional file 4 and Additional file 5**.** There are five aspects that explain the sources of risk of bias. First, many studies with > 5% of data missing increased the risk of bias. Second, appropriate analyses (eg, intention-to-treat analysis) were not used to estimate the effect of assignment to intervention, which threatened the deviations from intended interventions. Third, no blinding of assessors and it was difficult or impossible to blind participants in CGA interventions, so the measurement of subjective outcomes was with increased risk of bias. Fourth, one study was rated as a high risk of randomisation process due to uncertainty of random assignment and impossibility of allocation sequence concealment. Lastly, one study was rated as a high risk of selection of the reported result due to inconsistency with the pre-registered protocol.

### Results of meta-analysis

Meta-analysis results are summarized in Table 2.

**Table 2:** Summary of meta-analysis results

Outcomes	Group basis	Subgroup name	Number of analyses (number of studies)	Heterogeneity ***I***^***2***^(%)	Effect SMD/MD[95% CI]	***P*** value
Quality of life level	overall	/	15 (8)	49	0.12 [0.03, 0.21]	0.009
	Intervention model	CGA-team	6 (3)	44	0.05 [−0.08, 0.18]	0.46
		OAS	4 (2)	71	0.22 [−0.04, 0.48]	0.09
		CGA-unit	2 (1)	56	0.23 [−0.01, 0.46]	0.06
		HAS	2 (1)	21	0.17 [−0.04, 0.38]	0.11
		HHAS	1 (1)	/	0.00 [−0.23, 0.23]	1.00
	Mean age of participants	≤ 80 years	4 (2)	77	0.20 [−0.15, 0.55]	0.26
		> 80 years	11 (6)	32	0.11 [0.02, 0.19]	0.01
	Outcome measure time	≤ 3 months	7 (6)	48	0.16 [0.03, 0.29]	0.01
		3–12 months	1 (1)	/	0.02 [−0.19, 0.23]	0.83
		≥ 12 months	7 (5)	57	0.10 [−0.06, 0.25]	0.21
	Evaluation instruments	EQ-5D	8 (4)	0	0.05 [−0.03, 0.13]	0.24
		15D questionnaire	1 (1)	/	0.44 [0.14, 0.74]	0.004
		PGCMS	2 (1)	0	−0.06 [−0.26, 0.13]	0.53
		EQ-VAS	2 (1)	56	0.23 [−0.01, 0.46]	0.06
		GWB	2 (1)	0	0.53 [0.24, 0.81]	0.0003
Change of quality of life	overall	/	5 (3)	5	0.24 [0.10, 0.39]	0.0008
SF-36 Change values for physical functioning dimension	overall	/	5 (4)	0	0.11 [0.05, 0.16]	0.0002
SF-36 Change values for physical limitation dimension	overall	/	5 (4)	64	0.04 [−0.05, 0.14]	0.39
SF-36 Change values for general health dimension	overall	/	5 (4)	0	0.10 [0.05, 0.16]	0.0003
SF-36 Change values for body pain dimension	overall	/	5 (4)	56	0.09 [0.00, 0.18]	0.04
SF-36 Change values for mental health dimension	overall	/	5 (4)	22	0.13 [0.06, 0.19]	0.0001
SF-36 Change values for emotional limitation dimension	overall	/	5 (4)	0	0.04 [−0.02, 0.10]	0.17
SF-36 Change values for energy dimension	overall	/	5 (4)	65	0.15 [0.06, 0.25]	0.002
SF-36 Change values for social activity dimension	overall	/	5 (4)	37	0.10 [0.02, 0.17]	0.01
Caregiver burden	overall	/	2 (2)	35	−0.56 [−0.97, −0.15]	0.007
Length of hospital stay	overall	/	22 (17)	100	−1.04 [−3.57, 1.49]	0.42
	Intervention model	CGA-unit	9 (6)	88	0.60 [−1.01, 2.22]	0.46
		CGA-team	12 (10)	100	−1.59 [−5.23, 2.05]	0.39
		OAS	1 (1)	/	−4.10 [−8.73, 0.53]	0.08
	Mean age of participants	≤ 80 years	8 (7)	0	−0.24 [−0.57, 0.09]	0.16
		> 80 years	14 (10)	100	−0.85 [−4.17, 2.48]	0.62
	Outcome measure time	≤ 3 months	15 (14)	100	0.13 [−2.56, 2.82]	0.93
		3–12 months	1 (1)	/	3.70 [−3.55, 10.95]	0.32
		≥ 12 months	6 (5)	100	−5.17 [−12.35, 2.01]	0.16

SMD, standardised mean difference; MD, mean difference; CI, confidence interval; CGA, comprehensive geriatric assessment; OAS, Outpatient Assessment Service; HHAS, Hospital Home Assessment Service; HAS, Home Assessment Service; PGCMS, Philadelphia Geriatric Centre Morale Scale; EQ-VAS, EuroQoL-visual analog scale; GWB, Rand general well-being; SF-36, 36-Item Short Form Health Survey questionnaire

### Quality of life

The evaluation instruments for the included studies are shown in Table 1.

#### Quality of life level

**Fig. 3:**
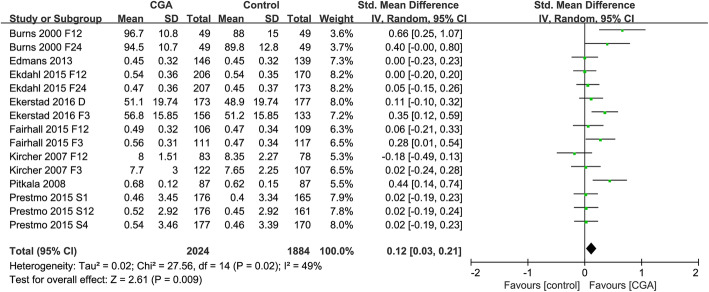
Forest plot of the effect of CGA intervention on quality of life level

Eight studies were included in the meta-analysis to evaluate the impact of CGA interventions on the quality of life levels of older people [35–37, 46, 49, 51, 54, 55]. Meta-analysis results showed that CGA interventions improved the quality of life of older people (SMD = 0.12; 95% CI = 0.03 to 0.21; *P* = 0.009). (Fig. 3) There was moderate heterogeneity (*P* = 0.02, *I*^*2*^ = 49%).

Based on the funnel plot asymmetry, evidence of publication bias was found (Additional file 6). Based on Egger’s test (*P* = 0.029), evidence of small-study effect was found (Additional file 6). Since there was evidence of small-study effect, we performed a sensitivity analysis excluding data from Burns 2000 and Piktala 2008. The results unveiled a trend of significant effect in quality of life (SMD = 0.07; 95% CI = − 0.00 to 0.13; *P* = 0.05), and decreased between-study heterogeneity from 49 to 10%. Furthermore, sensitivity analysis revealed that the direction of the main result were largely unchanged by excluding studies by turns (Additional file 7). However, sensitivity analysis only including studies with low risk of measurement of the outcome changes the direction of the main result (Additional file 8).

Table 2 summarizes the results of subgroup analyses. The overall effect on each of the subgroups (CGA-team [35–37], CGA-unit [46], OAS [49, 51], HHAS [55], HAS [54]) did not exert a significant effect on the quality of life, but reflected a trend in favour of CGA interventions, with SMD > 0. (Additional file 9). The subgroup analysis did not substantially explain or reduce the heterogeneity.

Subgroup analysis was also performed according to the participants’ age (≤ 80 years and > 80 years). We found that CGA intervention was superior to usual care for the quality of life only in subgroup of participants age > 80 years [36, 37, 46, 51, 54, 55] (Additional file 10).

When we grouped the studies according to the outcome measure time (at follow-up / after surgery ≤ 3 months, 3–12 months, ≥ 12 months), we found that CGA intervention was superior to usual care for the quality of life only at follow-up ≤ 3 months (Additional file 11).

When the studies were grouped according to evaluation instruments (EQ-5D、EQ-VAS、15 D questionnaire、PGCMS, GWB), we found that there was a significant difference between the subgroups(*P* = 0.0008, *I*^*2*^ = 78.8%). No differences in the scores of quality of life measured by EQ-5D [37, 51, 54, 55], PGCMS [35] and EQ-VAS [46] were found between CGA intervention and usual care. CGA intervention demonstrated benefit for the quality of life when measured by 15D questionnaire [36] and the Rand general well-being (GWB) inventory [49] (Additional file 12).

#### Change of quality of life

The meta-analysis of the change of quality of life included three studies [36, 49, 54]. The results of the meta-analysis showed a positive effect of the CGA intervention on quality of life (SMD = 0.24; 95% CI = 0.10 to 0.39; *P =* 0.0008), with negligible heterogeneity (*P* = 0.38, *I*^*2*^ = 5%).

#### SF-36 change values for each dimension

The SF-36 does not evaluate the general quality of life. Thus, the eight SF-36 domains (physical functioning, physical limitation, general health, body pain, mental health, emotional limitation, energy and social activity) were analysed separately [56]. A total of 4 studies were included and meta-analyses were conducted on the change values of each dimension of the SF-36 [44, 48, 53]. With the exception of the physical limitations and emotional limitations dimensions, the change values for other six dimensions were significantly better in the CGA intervention group than in the usual care group, and the specific change values are shown in the Table 2.

### Caregiver burden

**Fig. 4:**
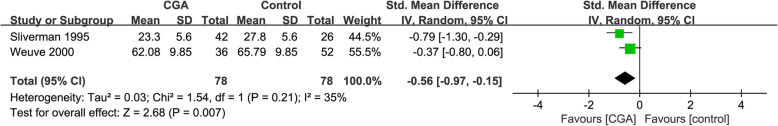
Forest plot of the effect of CGA intervention on caregiver burden

Meta-analysis results indicated that the CGA intervention reduced caregiver burden (SMD = − 0.56; 95% CI = − 0.97 to − 0.15; *P* = 0.007), with low heterogeneity between studies (*P* = 0.21, *I*^*2*^ = 35%) (Fig. 4).

### Length of hospital stay

**Fig. 5:**
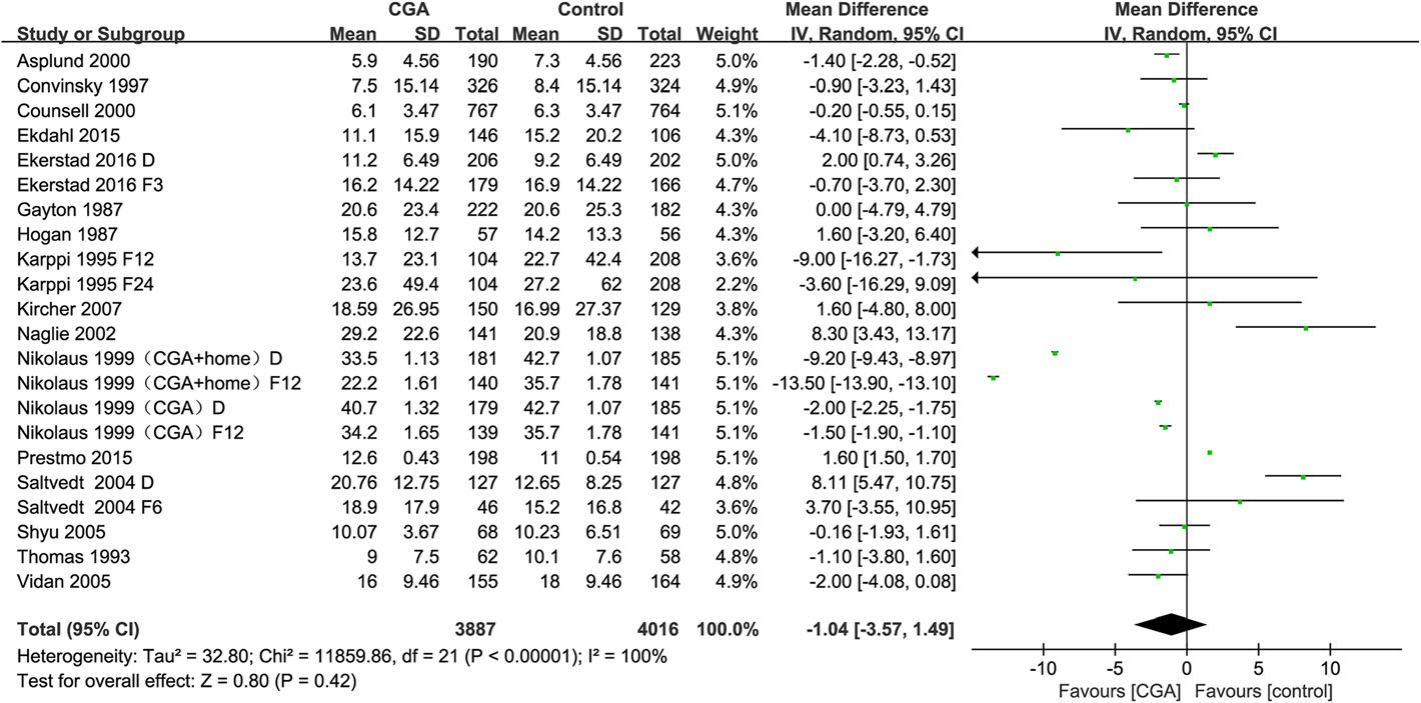
Forest plot of the effect of CGA intervention on length of hospital stay

Result of meta-analysis indicated that there was no benefit from the CGA intervention on length of hospital stay (MD = − 1.04; 95% CI = − 3.57 to 1.49; *P* = 0.42), with high heterogeneity between studies (*P* < 0.00001, *I*^*2*^ = 100%) (Fig. 5). The heterogeneity was so remarkable (*P* < 0.00001, *I*^*2*^ = 100%) that it could not be altered by omitting any single study from the sensitivity analysis. Sensitivity analysis revealed that the direction of the main association were largely unchanged neither by only including studies with some concerns in risk of bias nor by excluding studies by turns (Additional files 8 and 13). Based on the funnel plot asymmetry, no evidence of publication bias was found (Additional file 14). Based on Egger’s test (*P* = 0.388), no evidence of small-study effect was found (Additional file 14).

The overall effect on each of the subgroups based on intervention model, participants' age or outcome measure time did not reveal a significant difference between the CGA intervention and usual care (Table 2).

## Discussion

To our knowledge, this is the first meta-analysis that extensively included randomised controlled trials of different CGA intervention models to explore the effectiveness of CGA interventions on the quality of life and caregiver burden. In addition, this study also explored the effect of the CGA intervention on the length of hospital stay. The meta-analyses of the included studies showed that CGA interventions improved the quality of life and reduced the caregiver burden compared to usual care, but had a similar impact on the length of hospital stay as usual care.

Only Ekdahl et al. has conducted a meta-analysis of the impact of CGA interventions on quality of life, and its results are consistent and inconsistent with the results of this study [14]. It included five related RCTs published before 2007, and the results of the subgroup analysis (CGA-team and CGA-unit subgroup) as well as the overall combined effect showed no effect of the CGA intervention on quality of life in hospitalised frail older people. This is consistent with our results of subgroup analysis based on the CGA intervention model, but inconsistent with the overall combined effect. There are two main reasons for the inconsistency in the overall combined effect: firstly, the number of studies included in our meta-analysis was 8 (with 15 analyses), and the included studies contained studies published after 2007. The feasibility and acceptability of recent CGA intervention studies was better as the CGA intervention protocol was refined; when excluding related RCTs published before 2007, positive results in terms of improved quality of life were still found (SMD = 0.09; 95% CI = 0.01to 0.17; *P* = 0.03), while heterogeneity was reduced (*I*^*2*^ = 33%). Second, our subgroup analysis showed that there was no statistical difference in the effect of the 5 CGA intervention models versus usual care on quality of life, but the SMD was greater than 0 for all 4 subgroups except for the HHAS subgroup (SMD = 0), suggesting a trend towards improved quality of life in all 4 subgroups [57]. However, the SMD for 2 subgroups of Ekdahl et al. was less than 0. Therefore, the trend towards positive outcomes for the 4 subgroups in our study contributed to the overall positive results. As health professionals, we are concerned about the effects of CGA interventions and also the circumstances in which positive CGA intervention effects are more likely to be achieved. This study found that positive effects of CGA interventions (quality of life improvements) were more likely to occur in the following situations: firstly, only when participants were older than 80 years, CGA interventions improved their quality of life. However, as there were only 2 studies in the subgroup of participants aged ≤80 years, there may have been insufficient data for chance results. Previous systematic review suggested that the effect of age differences on the effectiveness of CGA interventions is uncertain and needs to be further explored in future studies [8]. Second, CGA interventions improved the quality of life of older people only at follow up ≤3 months. Functional status is the most significant predictor of quality of life [58, 59], and the focus of comprehensive geriatric assessment, and different CGA intervention models include appropriate rehabilitation interventions and multidisciplinary interventions to maintain the patient’s function [9]. A meta-analysis showed that the effect of multidisciplinary inpatient rehabilitation interventions to improve function in older people only occurred in the short term and the effect disappeared at 3–12 months follow-up [16], suggesting that the positive results of multidisciplinary inpatient rehabilitation interventions on quality of life may only occur in the short term, which supports the findings of this study to some extent. Third, the selection of instruments with high sensitivity to changes in quality of life in older people contributed to the positive results observed. The most used scale in the studies included in this review was the EQ-5D, followed by the SF-36. No differences in the scores of quality of life measured by EQ-5D were found between CGA intervention and usual care, which were inconsistent with the results of the meta-analysis of overall quality of life levels as well as change of quality of life. This may be possibly related to the limited reflection [60], poor responsiveness and sensitivity of EQ-5D to quality of life of older adults, which was found in previous studies [54, 61]. Previous studies found some evidence that SF-36 was sensitive to changes in the quality of life of older people [59, 62]. Our meta-analysis of SF-36 change values for each dimension found that the CGA intervention was able to improve quality of life in older adults across six dimensions. To facilitate future meta-analyses to further clarify the impact of CGA interventions on quality of life, it is recommended that the SF-36 be used consistently when evaluating the effects of CGA interventions. Previous evidence suggested that caregiver burden can lead to reduced quality of life for caregiver, increased risk of caregiver death and increased risk of institutionalisation of older people [63, 64]. There has been concern about how caregiver burden changes after CGA interventions [65]. Our result of meta-analysis showed that CGA interventions reduced caregiver burden compared to usual care. This may be related to the fact that the CGA intervention in the 2 RCTs included in the meta-analysis contained supportive strategies for caregivers (providing recommendations and consultation regarding caregiving) [47, 50]. Silliman et al. [66] found that providing recommendations and family education for caregivers of older people was beneficial to their general health. Previous research suggested that providing supportive caregiver education can improve not only the quality of life of the caregiver, but also potentially the quality of life of the patient [67]. Therefore, providing supportive strategies for caregivers should be an essential part of CGA interventions in order to discover the greater benefits of CGA interventions. The results of this review showed that the CGA intervention had no significant effect on the length of hospital stay. The results of previous reviews are controversial, with some studies having similar results to ours [19, 20] and others inconsistent [17, 18]. The results of the sensitivity analysis suggested that our results are robust and stable, but due to the presence of high heterogeneity (heterogeneity was not reduced by subgroup analysis) and the controversial results of previous reviews, the strength of evidence for the results was limited. Therefore, future studies should further clarify the effect of CGA interventions on the length of hospital stay.

Some limitations of this study should be mentioned. Firstly, the overall risk of bias among the included studies is generally “high” or “some concerns”, and caution is needed in the interpretation of the results. Second, there was moderate or even high heterogeneity between included studies, which persisted despite subgroup analyses; studies included in the overall quality of life level meta-analysis had publication bias, and its sensitive analyses that included only studies with low risk of measurement of outcome were inconsistent with the overall combined results, so its meta-analysis results may not be robust. Third, there were fewer included studies for the caregiver burden meta-analysis, so the strength of evidence for the results was limited. Fourth, the applicability of the results of this review is limited by the fact that the studies included in this review were mainly located in in European and American countries, with few studies from other regions. Fifth, as the included studies were limited to English-language published RCTs and did not include unpublished studies, there is a risk of increased publication bias.

## Conclusions

Compared to usual care, CGA intervention was effective in improving quality of life and reducing caregiver burden in older adults, but had no effect on the length of hospital stay. It is recommended that future studies apply the SF-36 to evaluate the impact of CGA interventions on quality of life and provide supportive strategies for caregivers as an essential part of the CGA intervention, with a view to finding additional benefits of CGA interventions.

## Supplementary Information

**Additional file 1.** PRISMA 2009 Checklist.

**Additional file 2.** Search strategies of the three databases.

**Additional file 3.** Characteristics and summary findings of included studies.

**Additional file 4. **Risk of bias of included studies about the outcome indicator caregiver burden.

**Additional file 5. **Risk of bias of included studies about the outcome indicator length of hospital stay.

**Additional file 6. **Publication bias test results of the quality of life.

**Additional file 7. **Sensitivity analysis for the quality of life by excluding studies by turns.

**Additional file 8.** Sensitivity analyses for primary outcome.

**Additional file 9. **Quality of life-subgroup analysis based on different intervention models.

**Additional file 10.** Quality of life-subgroup analysis based on participants' age**. **

**Additional file 11. **Quality of life-subgroup analysis based on outcome measure time.

**Additional file 12. **Quality of life-subgroup analysis based on evaluation instrument.

**Additional file 13. **Sensitivity analysis for length of hospital stay by excluding studies by turns**.**

**Additional file 14.** Publication bias test results of the length of hospital stay.

## Data Availability

The datasets supporting the conclusions of this article are included within the article and its additional files.

## Abbreviations

CGA: comprehensive geriatric assessment
RCTs: randomised controlled trials
SMD: standardised mean difference
MD: mean difference
CIs: confidence intervals
CI: confidence interval
SF-36: 36-Item Short Form Health Survey questionnaire
WHO: World Health Organization
RevMan: Review Manager
PRISMA: Preferred Reporting Items for Systematic Reviews and Meta-Analyses
OAS: outpatient assessment service
HHAS: hospital home assessment service
HAS: home assessment service
SD: standard deviations
IG: intervention group (comprehensive geriatric assessment intervention group)
CG: control group (usual care group)
EQ-VAS: EuroQoL-visual analog scale
PGCMS: Philadelphia Geriatric Centre Morale Scale
GWB: Rand general well-being inventory
GLS: perceived global life satisfaction
CES-D: Center for Epidemiologic Studies-Depression scale
ICECAP-O: ICEpop CA Pability measure for older people
DL: Depression List
D: discharge
F3: at follow up 3 months
F6: at follow up 6 months
F12: at follow up 12 months
F24: at follow up 24 months
S1: 1 month after surgery
S4: 4 month after surgery
S12: 12 month after surgery
R12: 12 months after randomisation
