# Supplementary material for: Effectiveness of comprehensive geriatric assessment intervention on quality of life, caregiver burden and length of hospital stay: a systematic review and meta-analysis of randomised controlled trials
Source: BMC Geriatr. 2021 Jun 21;21:377. doi: 10.1186/s12877-021-02319-2 (PMC8218512; doi:10.1186/s12877-021-02319-2)
Supplement: Supplementary file 2 — Additional file 2. Search strategies of the three databases. [file 12877_2021_2319_MOESM2_ESM.doc]

**Additional file 2** Search strategies of the three database**s**

| **Database** | **Search terms** | **Results** |
| --- | --- | --- |
| **PubMed** |  |  |
|  | 1. "Aged"[Mesh] | [3,133,423](https://pubmed.ncbi.nlm.nih.gov/?term=) |
|  | 1. geriatric[Title/Abstract] | [42,021](https://pubmed.ncbi.nlm.nih.gov/?term=geriatric%5bTitle/Abstract%5d&filter=dates.1600/1/1-2020/2/29&size=100&ac=no&sort=relevance) |
|  | 1. elderly[Title/Abstract] | [243,648](https://pubmed.ncbi.nlm.nih.gov/?term=elderly%5bTitle/Abstract%5d&filter=dates.1600/1/1-2020/2/29&size=100&ac=no&sort=relevance) |
|  | 1. old[Title/Abstract] | [1,029,043](https://pubmed.ncbi.nlm.nih.gov/?term=old%5bTitle/Abstract%5d&filter=dates.1600/1/1-2020/2/29&size=100&ac=no&sort=relevance) |
|  | 1. gerontal[Title/Abstract] | [15](https://pubmed.ncbi.nlm.nih.gov/?term=gerontal%5bTitle/Abstract%5d&size=100&ac=no&sort=relevance) |
|  | 1. geratic[Title/Abstract] | 6 |
|  | 1. senior[Title/Abstract] | [32,381](https://pubmed.ncbi.nlm.nih.gov/?term=senior%5bTitle/Abstract%5d&filter=dates.1600/1/1-2020/2/29&size=100&ac=no&sort=relevance) |
|  | 1. elder[Title/Abstract]) | [8,326](https://pubmed.ncbi.nlm.nih.gov/?term=elder%5bTitle/Abstract%5d&filter=dates.1600/1/1-2020/2/29&size=100&ac=no&sort=relevance) |
|  | 1. {OR 1-8} | [4,070,202](https://pubmed.ncbi.nlm.nih.gov/?term=((((((() |
|  | 1. consultation[Title/Abstract] | [58,817](https://pubmed.ncbi.nlm.nih.gov/?term=consultation%5bTitle/Abstract%5d&filter=dates.1600/1/1-2020/2/29&size=100&ac=no&sort=relevance) |
|  | 1. evaluation[Title/Abstract] | [1,170,381](https://pubmed.ncbi.nlm.nih.gov/?term=evaluation%5bTitle/Abstract%5d&filter=dates.1600/1/1-2020/2/29&size=100&ac=no&sort=relevance) |
|  | 1. assess?[Title/Abstract] | [959,506](https://pubmed.ncbi.nlm.nih.gov/?term=assess?%5bTitle/Abstract%5d&filter=dates.1600/1/1-2020/2/29&size=100&ac=no&sort=relevance) |
|  | 1. {OR 10-12} | [2,083,667](https://pubmed.ncbi.nlm.nih.gov/?term=((assess?%5bTitle/Abstract%5d+AND+(1600/1/1:2020/2/29%5bpdat%5d))+OR+(consultation%5bTitle/Abstract%5d+AND+(1600/1/1:2020/2/29%5bpdat%5d)))+OR+(evaluation%5bTitle/Abstract%5d+AND+(1600/1/1:2020/2/29%5bpdat%5d))&filter=dates.1600/1/1-2020/2/29&size=100&ac=no&sort=relevance) |
|  | 1. interdisciplinary[Title/Abstract] | [36,032](https://pubmed.ncbi.nlm.nih.gov/?term=interdisciplinary%5bTitle/Abstract%5d&filter=dates.1600/1/1-2020/2/29&size=100&ac=no&sort=relevance) |
|  | 1. multifactorial[Title/Abstract] | [42,322](https://pubmed.ncbi.nlm.nih.gov/?term=multifactorial%5bTitle/Abstract%5d&filter=dates.1600/1/1-2020/2/29&size=100&ac=no&sort=relevance) |
|  | 1. multidimensional[Title/Abstract] | [38,389](https://pubmed.ncbi.nlm.nih.gov/?term=multidimensional%5bTitle/Abstract%5d&filter=dates.1600/1/1-2020/2/29&size=100&ac=no&sort=relevance) |
|  | 1. multidisciplinary[Title/Abstract] | [87,195](https://pubmed.ncbi.nlm.nih.gov/?term=multidisciplinary%5bTitle/Abstract%5d&filter=dates.1600/1/1-2020/2/29&size=100&ac=no&sort=relevance) |
|  | 1. {OR 14-17} | [199,665](https://pubmed.ncbi.nlm.nih.gov/?term=(((multidisciplinary%5bTitle/Abstract%5d+AND+(1600/1/1:2020/2/29%5bpdat%5d))+OR+(multidimensional%5bTitle/Abstract%5d+AND+(1600/1/1:2020/2/29%5bpdat%5d)))+OR+(multifactorial%5bTitle/Abstract%5d+AND+(1600/1/1:2020/2/29%5bpdat%5d)))+OR+(interdisciplinary%5bTitle/Abstract%5d+AND+(1600/1/1:2020/2/29%5bpdat%5d))&filter=dates.1600/1/1-2020/2/29&size=100&ac=no&sort=relevance) |
|  | 1. {9 AND 13 AND 18} | [8,530](https://pubmed.ncbi.nlm.nih.gov/?term=((((((interdisciplinary%5bTitle/Abstract%5d)+OR+(multifactorial%5bTitle/Abstract%5d))+OR+(multidimensional%5bTitle/Abstract%5d)))+OR+(multidisciplinary%5bTitle/Abstract%5d))+AND+(((consultation%5bTitle/Abstract%5d)+OR+(evaluation%5bTitle/Abstract%5d))+OR+(assess?%5bTitle/Abstract%5d)))+AND+(((((((() |
|  | 1. "Geriatric Assessment"[Mesh] | [27,792](https://pubmed.ncbi.nlm.nih.gov/?term=) |
|  | 1. "Health Services for the Aged"[Mesh] | [17,718](https://pubmed.ncbi.nlm.nih.gov/?term=) |
|  | 1. Assessment, Geriatric[Title/Abstract] | [108](https://pubmed.ncbi.nlm.nih.gov/?term=Assessment,+Geriatric%5bTitle/Abstract%5d&filter=dates.1600/1/1-2020/2/29&size=100&ac=no&sort=relevance) |
|  | 1. Assessments, Geriatric[Title/Abstract] | [1](https://pubmed.ncbi.nlm.nih.gov/?term=Assessments,+Geriatric%5bTitle/Abstract%5d&filter=dates.1600/1/1-2020/2/29&size=100&ac=no&sort=relevance) |
|  | 1. Geriatric Assessments[Title/Abstract] | [243](https://pubmed.ncbi.nlm.nih.gov/?term=Geriatric+Assessments%5bTitle/Abstract%5d&filter=dates.1600/1/1-2020/2/29&size=100&ac=no&sort=relevance) |
|  | 1. Geriatric Health Services[Title/Abstract] | [57](https://pubmed.ncbi.nlm.nih.gov/?term=Geriatric+Health+Services%5bTitle/Abstract%5d&filter=dates.1600/1/1-2020/2/29&size=100&ac=no&sort=relevance) |
|  | 1. Geriatric Health Service[Title/Abstract] | [22](https://pubmed.ncbi.nlm.nih.gov/?term=Geriatric+Health+Service%5bTitle/Abstract%5d&filter=dates.1600/1/1-2020/2/29&size=100&ac=no&sort=relevance) |
|  | 1. Health Services for the Elderly[Title/Abstract] | [92](https://pubmed.ncbi.nlm.nih.gov/?term=Health+Services+for+the+Elderly%5bTitle/Abstract%5d&filter=dates.1600/1/1-2020/2/29&size=100&ac=no&sort=relevance) |
|  | 1. Health Services, Geriatric[Title/Abstract] | [9](https://pubmed.ncbi.nlm.nih.gov/?term=Health+Services,+Geriatric%5bTitle/Abstract%5d&filter=dates.1600/1/1-2020/2/29&size=100&ac=no&sort=relevance) |
|  | 1. Health Service, Geriatric[Title/Abstract] | [3](https://pubmed.ncbi.nlm.nih.gov/?term=Health+Service,+Geriatric%5bTitle/Abstract%5d&filter=dates.1600/1/1-2020/2/29&size=100&ac=no&sort=relevance) |
|  | 1. Service, Geriatric Health[Title/Abstract] | [221](https://pubmed.ncbi.nlm.nih.gov/?term=Service,+Geriatric+Health%5bTitle/Abstract%5d&filter=dates.1600/1/1-2020/2/29&size=100&ac=no&sort=relevance) |
|  | 1. Services, Geriatric Health[Title/Abstract] | [221](https://pubmed.ncbi.nlm.nih.gov/?term=Services,+Geriatric+Health%5bTitle/Abstract%5d&filter=dates.1600/1/1-2020/2/29&size=100&ac=no&sort=relevance) |
|  | 1. Health Services for Aged[Title/Abstract] | [5](https://pubmed.ncbi.nlm.nih.gov/?term=Health+Services+for+Aged%5bTitle/Abstract%5d&filter=dates.1600/1/1-2020/2/29&size=100&ac=no&sort=relevance) |
|  | 1. comprehensive geriatric assessment[Title/Abstract] | [2,014](https://pubmed.ncbi.nlm.nih.gov/?term=Comprehensive+geriatric+Assessment%5bTitle/Abstract%5d&filter=dates.1600/1/1-2020/2/29&size=100&ac=no&sort=relevance) |
|  | 1. geriatric evaluation management unit[Title/Abstract] | [38](https://pubmed.ncbi.nlm.nih.gov/?term=geriatric+evaluation+management+unit%5bTitle/Abstract%5d&filter=dates.1600/1/1-2020/2/29&size=100&ac=no&sort=relevance) |
|  | 1. acute care for elders unit[Title/Abstract] | [23](https://pubmed.ncbi.nlm.nih.gov/?term=acute+care+for+elders+unit%5bTitle/Abstract%5d&filter=dates.1600/1/1-2020/2/29&size=100&ac=no&sort=relevance) |
|  | 1. inpatient geriatric consultation service[Title/Abstract] | [4](https://pubmed.ncbi.nlm.nih.gov/?term=inpatient+geriatric+consultation+service%5bTitle/Abstract%5d&filter=dates.1600/1/1-2020/2/29&size=100&ac=no&sort=relevance) |
|  | 1. mobile acute care of the elderly[Title/Abstract] | [2](https://pubmed.ncbi.nlm.nih.gov/?term=mobile+acute+care+of+the+elderly%5bTitle/Abstract%5d&filter=dates.1600/1/1-2020/2/29&size=100&ac=no&sort=relevance) |
|  | 1. home assessment service[Title/Abstract] | [33](https://pubmed.ncbi.nlm.nih.gov/?term=home+assessment+service%5bTitle/Abstract%5d&filter=dates.1600/1/1-2020/2/29&size=100&ac=no&sort=relevance) |
|  | 1. hospital home assessment service[Title/Abstract] | [18](https://pubmed.ncbi.nlm.nih.gov/?term=hospital+home+assessment+service%5bTitle/Abstract%5d&filter=dates.1600/1/1-2020/2/29&size=100&ac=no&sort=relevance) |
|  | 1. outpatient assessment service[Title/Abstract] | [67](https://pubmed.ncbi.nlm.nih.gov/?term=outpatient+assessment+service%5bTitle/Abstract%5d&filter=dates.1600/1/1-2020/2/29&size=100&ac=no&sort=relevance) |
|  | 1. posthospital discharge CGA[Title/Abstract] | [4](https://pubmed.ncbi.nlm.nih.gov/?term=posthospital+discharge+CGA%5bTitle/Abstract%5d&filter=dates.1600/1/1-2020/2/29&size=100&ac=no&sort=relevance) |
|  | 1. multidimensional geriatric assessment[Title/Abstract] | [83](https://pubmed.ncbi.nlm.nih.gov/?term=multidimensional+geriatric+Assessment%5bTitle/Abstract%5d&filter=dates.1600/1/1-2020/2/29&size=100&ac=no&sort=relevance) |
|  | 1. in-home CGA[Title/Abstract] | [7](https://pubmed.ncbi.nlm.nih.gov/?term=in-home+CGA%5bTitle/Abstract%5d&filter=dates.1600/1/1-2020/2/29&size=100&ac=no&sort=relevance) |
|  | 1. outpatient CGA[Title/Abstract] | [10](https://pubmed.ncbi.nlm.nih.gov/?term=outpatient+CGA%5bTitle/Abstract%5d&filter=dates.1600/1/1-2020/2/29&size=100&ac=no&sort=relevance) |
|  | 1. home-based CGA[Title/Abstract] | [1](https://pubmed.ncbi.nlm.nih.gov/?term=home-based+CGA%5bTitle/Abstract%5d&filter=dates.1600/1/1-2020/2/29&size=100&ac=no&sort=relevance) |
|  | 1. hospital-based CGA[Title/Abstract] | [7](https://pubmed.ncbi.nlm.nih.gov/?term=hospital-based+CGA%5bTitle/Abstract%5d&filter=dates.1600/1/1-2020/2/29&size=100&ac=no&sort=relevance) |
|  | 1. inpatient CGA[Title/Abstract] | [4](https://pubmed.ncbi.nlm.nih.gov/?term=inpatient+CGA%5bTitle/Abstract%5d&filter=dates.1600/1/1-2020/2/29&size=100&ac=no&sort=relevance) |
|  | 1. geriatric unit[Title/Abstract] | [550](https://pubmed.ncbi.nlm.nih.gov/?term=geriatric+unit%5bTitle/Abstract%5d&filter=dates.1600/1/1-2020/2/29&size=100&ac=no&sort=relevance) |
|  | 1. specialist geriatric[Title/Abstract] | [43](https://pubmed.ncbi.nlm.nih.gov/?term=specialist+geriatric%5bTitle/Abstract%5d&filter=dates.1600/1/1-2020/2/29&size=100&ac=no&sort=relevance) |
|  | 1. acute geriatric[Title/Abstract] | [562](https://pubmed.ncbi.nlm.nih.gov/?term=acute+geriatric%5bTitle/Abstract%5d&filter=dates.1600/1/1-2020/2/29&size=100&ac=no&sort=relevance) |
|  | 1. ACE units[Title/Abstract] | [17](https://pubmed.ncbi.nlm.nih.gov/?term=ACE+units%5bTitle/Abstract%5d&filter=dates.1600/1/1-2020/2/29&size=100&ac=no&sort=relevance) |
|  | 1. GEMU[Title/Abstract] | [30](https://pubmed.ncbi.nlm.nih.gov/?term=GEMU%5bTitle/Abstract%5d&filter=dates.1600/1/1-2020/2/29&size=100&ac=no&sort=relevance) |
|  | 1. IGCS[Title/Abstract] | [166](https://pubmed.ncbi.nlm.nih.gov/?term=IGCS%5bTitle/Abstract%5d&filter=dates.1600/1/1-2020/2/29&size=100&ac=no&sort=relevance) |
|  | 1. HAS[Title/Abstract] | [4,694](https://pubmed.ncbi.nlm.nih.gov/?term=HAS%5bTitle/Abstract%5d&filter=dates.1600/1/1-2020/2/29&size=100&ac=no&sort=relevance) |
|  | 1. HHAS[Title/Abstract] | [178](https://pubmed.ncbi.nlm.nih.gov/?term=HHAS%5bTitle/Abstract%5d&filter=dates.1600/1/1-2020/2/29&size=100&ac=no&sort=relevance) |
|  | 1. {OR 20-55} | [50,613](https://pubmed.ncbi.nlm.nih.gov/?term=longqueryd8c23f2827c8bd27645f&filter=dates.1600/1/1-2020/2/29&size=100&ac=no&sort=relevance) |
|  | 1. 19 OR 56 | [58,380](https://pubmed.ncbi.nlm.nih.gov/?term=longqueryd580e716d597021d0717&filter=dates.1600/1/1-2020/2/29&size=100&ac=no&sort=relevance) |
|  | 1. "Quality of Life"[Mesh] | [196,245](https://pubmed.ncbi.nlm.nih.gov/?term=(() |
|  | 1. Life Quality[Title/Abstract] | [6,987](https://pubmed.ncbi.nlm.nih.gov/?term=Life+Quality%5bTitle/Abstract%5d&filter=dates.1600/1/1-2020/2/29&size=100&ac=no&sort=relevance) |
|  | 1. Health-Related Quality Of Life[Title/Abstract] | [42,773](https://pubmed.ncbi.nlm.nih.gov/?term=Health-Related+Quality+Of+Life%5bTitle/Abstract%5d&filter=dates.1600/1/1-2020/2/29&size=100&ac=no&sort=relevance) |
|  | 1. Health Related Quality Of Life[Title/Abstract] | [42,773](https://pubmed.ncbi.nlm.nih.gov/?term=Health+Related+Quality+Of+Life%5bTitle/Abstract%5d&filter=dates.1600/1/1-2020/2/29&size=100&ac=no&sort=relevance) |
|  | 1. HRQOL[Title/Abstract] | [16,554](https://pubmed.ncbi.nlm.nih.gov/?term=HRQOL%5bTitle/Abstract%5d&filter=dates.1600/1/1-2020/2/29&size=100&ac=no&sort=relevance) |
|  | 1. Life quality[Title/Abstract] | [6,987](https://pubmed.ncbi.nlm.nih.gov/?term=Life+quality%5bTitle/Abstract%5d&filter=dates.1600/1/1-2020/2/29&size=100&ac=no&sort=relevance) |
|  | 1. Quality of living[Title/Abstract] | [151](https://pubmed.ncbi.nlm.nih.gov/?term=Quality+of+living%5bTitle/Abstract%5d&filter=dates.1600/1/1-2020/2/29&size=100&ac=no&sort=relevance) |
|  | 1. {OR 58-64} | [210,447](https://pubmed.ncbi.nlm.nih.gov/?term=Quality+of+living%5bTitle/Abstract%5d+OR+Life+quality%5bTitle/Abstract%5d+OR+HRQOL%5bTitle/Abstract%5d++OR+Health+Related+Quality+Of+Life%5bTitle/Abstract%5d++OR+Health-Related+Quality+Of+Life%5bTitle/Abstract%5dOR+Life+Quality%5bTitle/Abstract%5d+OR+) |
|  | 1. Pressure[Title/Abstract] | [807,546](https://pubmed.ncbi.nlm.nih.gov/?term=Pressure%5bTitle/Abstract%5d&filter=dates.1600/1/1-2020/2/29&size=100&ac=no&sort=relevance) |
|  | 1. Burden[Title/Abstract] | [189,473](https://pubmed.ncbi.nlm.nih.gov/?term=Burden%5bTitle/Abstract%5d&filter=dates.1600/1/1-2020/2/29&size=100&ac=no&sort=relevance) |
|  | 1. Stress[Title/Abstract] | [731,500](https://pubmed.ncbi.nlm.nih.gov/?term=Stress%5bTitle/Abstract%5d&filter=dates.1600/1/1-2020/2/29&size=100&ac=no&sort=relevance) |
|  | 1. Strain[Title/Abstract] | [426,130](https://pubmed.ncbi.nlm.nih.gov/?term=Strain%5bTitle/Abstract%5d&filter=dates.1600/1/1-2020/2/29&size=100&ac=no&sort=relevance) |
|  | 1. {OR 66-69} | [812,808](https://pubmed.ncbi.nlm.nih.gov/?term=Strain%5bTitle/Abstract%5d+OR+Stress%5bTitle/Abstract%5d+Or+Burden%5bTitle/Abstract%5d+OR+Pressure%5bTitle/Abstract%5d&filter=dates.1600/1/1-2020/2/29&size=100&ac=no&sort=relevance) |
|  | 1. Caregivers"[Mesh] | [36,986](https://pubmed.ncbi.nlm.nih.gov/?term=) |
|  | 1. Carers[Title/Abstract] | [11,978](https://pubmed.ncbi.nlm.nih.gov/?term=Carers%5bTitle/Abstract%5d&filter=dates.1600/1/1-2020/2/29&size=100&ac=no&sort=relevance) |
|  | 1. Carer[Title/Abstract] | [5,027](https://pubmed.ncbi.nlm.nih.gov/?term=Carer%5bTitle/Abstract%5d&filter=dates.1600/1/1-2020/2/29&size=100&ac=no&sort=relevance) |
|  | 1. Care Giver[Title/Abstract] | [715](https://pubmed.ncbi.nlm.nih.gov/?term=Care+Giver%5bTitle/Abstract%5d&filter=dates.1600/1/1-2020/2/29&size=100&ac=no&sort=relevance) |
|  | 1. Care Givers[Title/Abstract] | [2,047](https://pubmed.ncbi.nlm.nih.gov/?term=Care+Givers%5bTitle/Abstract%5d&filter=dates.1600/1/1-2020/2/29&size=100&ac=no&sort=relevance) |
|  | 1. Spouse Caregivers[Title/Abstract] | [281](https://pubmed.ncbi.nlm.nih.gov/?term=Spouse+Caregivers%5bTitle/Abstract%5d&filter=dates.1600/1/1-2020/2/29&size=100&ac=no&sort=relevance) |
|  | 1. Caregiver, Spouse[Title/Abstract] | [13](https://pubmed.ncbi.nlm.nih.gov/?term=Caregiver,+Spouse%5bTitle/Abstract%5d&filter=dates.1600/1/1-2020/2/29&size=100&ac=no&sort=relevance) |
|  | 1. Caregivers, Spouse[Title/Abstract] | [8](https://pubmed.ncbi.nlm.nih.gov/?term=Caregivers,+Spouse%5bTitle/Abstract%5d&filter=dates.1600/1/1-2020/2/29&size=100&ac=no&sort=relevance) |
|  | 1. Spouse Caregiver[Title/Abstract] | [69](https://pubmed.ncbi.nlm.nih.gov/?term=Spouse+Caregiver%5bTitle/Abstract%5d&filter=dates.1600/1/1-2020/2/29&size=100&ac=no&sort=relevance) |
|  | 1. Family Caregivers[Title/Abstract] | [6,426](https://pubmed.ncbi.nlm.nih.gov/?term=Family+Caregivers%5bTitle/Abstract%5d&filter=dates.1600/1/1-2020/2/29&size=100&ac=no&sort=relevance) |
|  | 1. Caregiver, Family[Title/Abstract] | [76](https://pubmed.ncbi.nlm.nih.gov/?term=Caregiver,+Family%5bTitle/Abstract%5d&filter=dates.1600/1/1-2020/2/29&size=100&ac=no&sort=relevance) |
|  | 1. Caregivers, Family[Title/Abstract] | [138](https://pubmed.ncbi.nlm.nih.gov/?term=Caregivers,+Family%5bTitle/Abstract%5d&filter=dates.1600/1/1-2020/2/29&size=100&ac=no&sort=relevance) |
|  | 1. Family Caregiver[Title/Abstract] | [1,613](https://pubmed.ncbi.nlm.nih.gov/?term=Family+Caregiver%5bTitle/Abstract%5d&filter=dates.1600/1/1-2020/2/29&size=100&ac=no&sort=relevance) |
|  | 1. long-term care[Title/Abstract] | [20,651](https://pubmed.ncbi.nlm.nih.gov/?term=long-term+care%5bTitle/Abstract%5d&filter=dates.1600/1/1-2020/2/29&size=100&ac=no&sort=relevance) |
|  | 1. medical care[Title/Abstract] | [50,224](https://pubmed.ncbi.nlm.nih.gov/?term=medical+care%5bTitle/Abstract%5d&filter=dates.1600/1/1-2020/2/29&size=100&ac=no&sort=relevance) |
|  | 1. nursing care[Title/Abstract] | [25,821](https://pubmed.ncbi.nlm.nih.gov/?term=nursing+care%5bTitle/Abstract%5d&filter=dates.1600/1/1-2020/2/29&size=100&ac=no&sort=relevance) |
|  | 1. Care provider[Title/Abstract] | [11,089](https://pubmed.ncbi.nlm.nih.gov/?term=Care+provider%5bTitle/Abstract%5d&filter=dates.1600/1/1-2020/2/29&size=100&ac=no&sort=relevance) |
|  | 1. nursing Provider[Title/Abstract] | [21](https://pubmed.ncbi.nlm.nih.gov/?term=Nursing+Provider%5bTitle/Abstract%5d&filter=dates.1600/1/1-2020/2/29&size=100&ac=no&sort=relevance) |
|  | 1. {OR71-88} | [153,968](https://pubmed.ncbi.nlm.nih.gov/?term=) |
|  | 1. 70 AND 89 | [4,537](https://pubmed.ncbi.nlm.nih.gov/?term=() |
|  | 1. "length of stay"[Mesh] | [88,779](https://pubmed.ncbi.nlm.nih.gov/?term=) |
|  | 1. stay length[Title/Abstract] | [548](https://pubmed.ncbi.nlm.nih.gov/?term=stay+length%5bTitle/Abstract%5d&sort=&filter=dates.1600/1/1-2020/2/29&size=100) |
|  | 1. stay lengths[Title/Abstract] | [26](https://pubmed.ncbi.nlm.nih.gov/?term=stay+lengths%5bTitle/Abstract%5d&filter=dates.1600/1/1-2020/2/29&size=100&ac=no&sort=relevance) |
|  | 1. hospital stay[Title/Abstract] | [73,825](https://pubmed.ncbi.nlm.nih.gov/?term=hospital+stay%5bTitle/Abstract%5d&filter=dates.1600/1/1-2020/2/29&size=100&ac=no&sort=relevance) |
|  | 1. hospital stays[Title/Abstract] | [8,213](https://pubmed.ncbi.nlm.nih.gov/?term=hospital+stays%5bTitle/Abstract%5d&filter=dates.1600/1/1-2020/2/29&size=100&ac=no&sort=relevance) |
|  | 1. stay, hospital[Title/Abstract] | [899](https://pubmed.ncbi.nlm.nih.gov/?term=stay,+hospital%5bTitle/Abstract%5d&filter=dates.1600/1/1-2020/2/29&size=100&ac=no&sort=relevance) |
|  | 1. stays, hospital[Title/Abstract] | [26](https://pubmed.ncbi.nlm.nih.gov/?term=stays,+hospital%5bTitle/Abstract%5d&filter=dates.1600/1/1-2020/2/29&size=100&ac=no&sort=relevance) |
|  | 1. length of hospital stay[Title/Abstract] | [21,642](https://pubmed.ncbi.nlm.nih.gov/?term=length+of+hospital+stay%5bTitle/Abstract%5d&filter=dates.1600/1/1-2020/2/29&size=100&ac=no&sort=relevance) |
|  | 1. hospital length of stay[Title/Abstract] | [8,456](https://pubmed.ncbi.nlm.nih.gov/?term=hospital+length+of+stay%5bTitle/Abstract%5d&filter=dates.1600/1/1-2020/2/29&size=100&ac=no&sort=relevance) |
|  | 1. {OR 91-99} | [145,429](https://pubmed.ncbi.nlm.nih.gov/?term=) |
|  | 1. 65 OR 90 OR 100 | [356,570](https://pubmed.ncbi.nlm.nih.gov/?term=((() |
|  | 1. 57 AND 101 | [6,365](https://pubmed.ncbi.nlm.nih.gov/?term=longquery3b6787285b8e3cee0105&filter=dates.1600/1/1-2020/2/29&size=100&ac=no&sort=relevance) |
|  | 1. "Randomized Controlled Trials as Topic"[Mesh] | [138,712](https://pubmed.ncbi.nlm.nih.gov/?term=) |
|  | 1. Clinical Trials, Randomized[Title/Abstract] | [151](https://pubmed.ncbi.nlm.nih.gov/?term=Clinical+Trials,+Randomized%5bTitle/Abstract%5d&filter=dates.1600/1/1-2020/2/29&size=100&ac=no&sort=relevance) |
|  | 1. Trials, Randomized Clinical[Title/Abstract] | [16](https://pubmed.ncbi.nlm.nih.gov/?term=Trials,+Randomized+Clinical%5bTitle/Abstract%5d&filter=dates.1600/1/1-2020/2/29&size=100&ac=no&sort=relevance) |
|  | 1. Controlled Clinical Trials, Randomized[Title/Abstract] | [17](https://pubmed.ncbi.nlm.nih.gov/?term=Controlled+Clinical+Trials,+Randomized%5bTitle/Abstract%5d&filter=dates.1600/1/1-2020/2/29&size=100&ac=no&sort=relevance) |
|  | 1. randomized[Title/Abstract] | [509,715](https://pubmed.ncbi.nlm.nih.gov/?term=randomized%5bTitle/Abstract%5d&filter=dates.1600/1/1-2020/2/29&size=100&ac=no&sort=relevance) |
|  | 1. randomly[Title/Abstract] | [329,365](https://pubmed.ncbi.nlm.nih.gov/?term=randomly%5bTitle/Abstract%5d&filter=dates.1600/1/1-2020/2/29&size=100&ac=no&sort=relevance) |
|  | 1. random？[Title/Abstract] | [266,295](https://pubmed.ncbi.nlm.nih.gov/?term=random%EF%BC%9F%5bTitle/Abstract%5d&filter=dates.1600/1/1-2020/2/29&size=100&ac=no&sort=relevance) |
|  | 1. Randomized Controlled Trial [Publication Type] | [513,695](https://pubmed.ncbi.nlm.nih.gov/?term=Randomized+Controlled+Trial%5bPublication+Type%5d&filter=dates.1600/1/1-2020/2/29&size=100&ac=no&sort=relevance) |
|  | 1. placebo[Title/Abstract] | [210,953](https://pubmed.ncbi.nlm.nih.gov/?term=placebo%5bTitle/Abstract%5d&filter=dates.1600/1/1-2020/2/29&size=100&ac=no&sort=relevance) |
|  | 1. single blind[Title/Abstract] | [13,446](https://pubmed.ncbi.nlm.nih.gov/?term=single+blind%5bTitle/Abstract%5d&filter=dates.1600/1/1-2020/2/29&size=100&ac=no&sort=relevance) |
|  | 1. double blind[Title/Abstract] | [137,891](https://pubmed.ncbi.nlm.nih.gov/?term=double+blind%5bTitle/Abstract%5d&filter=dates.1600/1/1-2020/2/29&size=100&ac=no&sort=relevance) |
|  | 1. triple blind[Title/Abstract] | [695](https://pubmed.ncbi.nlm.nih.gov/?term=triple+blind%5bTitle/Abstract%5d&filter=dates.1600/1/1-2020/2/29&size=100&ac=no&sort=relevance) |
|  | 1. {OR 103-114} | [1,321,496](https://pubmed.ncbi.nlm.nih.gov/?term=triple+blind%5bTitle/Abstract%5d+OR+double+blind%5bTitle/Abstract%5d+OR+single+blind%5bTitle/Abstract%5d+OR+placebo%5bTitle/Abstract%5d+OR+Randomized+Controlled+Trial%5bPublication+Type%5d+OR+random%EF%BC%9F%5bTitle/Abstract%5d+OR+randomly%5bTitle/Abstract%5d+OR+randomized%5bTitle/Abstract%5d+OR+Controlled+Clinical+Trials,+Randomized%5bTitle/Abstract%5d+OR+Trials,+Randomized+Clinical%5bTitle/Abstract%5d+OR+Clinical+Trials,+Randomized%5bTitle/Abstract%5d+OR+) |
|  | 1. 102 AND 115 | [**908**](https://pubmed.ncbi.nlm.nih.gov/?term=longquery926b104775b545427577&filter=dates.1600/1/1-2020/2/29&size=100&ac=no&sort=relevance) |
| **Embase** |  |  |
|  | 1. 'aged'/exp | 3,118,457 |
|  | 1. old:ab,ti | 1,508,955 |
|  | 1. elderly:ab,ti | 345,941 |
|  | 1. geriatric:ab,ti | 64,313 |
|  | 1. gerontal:ab,ti | 27 |
|  | 1. geratic:ab,ti | 8 |
|  | 1. senior:ab,ti | 47,144 |
|  | 1. elder:ab,ti | 12,685 |
|  | 1. older:ab,ti | 597,008 |
|  | 1. {OR 1-9} | 4,771,281 |
|  | 1. 'geriatric assessment'/exp | 16,575 |
|  | 1. 'elderly care'/exp | 77,476 |
|  | 1. 'geriatric care'/exp | 27,438 |
|  | 1. 'geriatric nursing'/exp | 12,892 |
|  | 1. 'assessment,geriatric':ab,ti | 155 |
|  | 1. 'assessments,geriatric':ab,ti | 3 |
|  | 1. 'geriatric assessments':ab,ti | 439 |
|  | 1. 'geriatric health services':ab,ti | 56 |
|  | 1. 'geriatric health service':ab,ti | 22 |
|  | 1. health services,geriatric':ab,ti | 1 |
|  | 1. 'health service,geriatric':ab,ti | 2 |
|  | 1. 'service,geriatric health':ab,ti | 0 |
|  | 1. 'services,geriatric health':ab,ti | 0 |
|  | 1. 'health services for aged':ab,ti | 3 |
|  | 1. 'comprehensive geriatric assessment':ab,ti | 3,681 |
|  | 1. 'geriatric evaluation and management unit':ab,ti | 46 |
|  | 1. acute care for elders unit':ab,ti | 38 |
|  | 1. 'inpatient geriatric consultation service':ab,ti | 2 |
|  | 1. 'mobile acute care of the elderly':ab,ti | 2 |
|  | 1. 'home assessment service':ab,ti | 0 |
|  | 1. 'hospital home assessment service':ab,ti | 0 |
|  | 1. 'outpatient assessment service':ab,ti | 0 |
|  | 1. 'posthospital discharge cga':ab,ti | 1 |
|  | 1. 'multidimensional geriatric assessment':ab,ti | 173 |
|  | 1. 'in-home cga':ab,ti | 6 |
|  | 1. 'outpatient cga':ab,ti | 15 |
|  | 1. 'home-based cga':ab,ti | 1 |
|  | 1. 'hospital-based cga':ab,ti | 0 |
|  | 1. 'inpatient cga':ab,ti | 7 |
|  | 1. 'geriatric unit':ab,ti | 983 |
|  | 1. 'specialist geriatric':ab,ti | 122 |
|  | 1. 'acute geriatric':ab,ti | 985 |
|  | 1. 'ace units':ab,ti | 40 |
|  | 1. 'gemu':ab,ti | 35 |
|  | 1. 'igcs':ab,ti | 2,114 |
|  | 1. 'hhas':ab,ti | 188 |
|  | 1. 'oas':ab,ti | 311,6 |
|  | 1. 'multidisciplinary':ab,ti | 136,022 |
|  | 1. 'interdisciplinary':ab,ti | 51,184 |
|  | 1. 'multifactorial':ab,ti | 56,292 |
|  | 1. 'multidimensional':ab,ti | 41,150 |
|  | 1. {OR 48-51} | 278,395 |
|  | 1. 'assess*':ab,ti | 4,216,034 |
|  | 1. 'evaluation':ab,ti | 1,674,324 |
|  | 1. 'consultation':ab,ti | 94,556 |
|  | 1. {OR 53-55} | 5,498,596 |
|  | 1. 56 AND 52 AND 10 | 22,663 |
|  | 1. {OR 11-47} | 98,365 |
|  | 1. 57 OR 58 | 118,459 |
|  | 1. 'caregiver'/exp | 81,151 |
|  | 1. 'long term care'/exp | 1,793,233 |
|  | 1. 'carer':ab,ti | 7,798 |
|  | 1. 'medical care'/exp | 1,091,467 |
|  | 1. 'nursing care'/exp | 38,944 |
|  | 1. 'care provider':ab,ti | 15,920 |
|  | 1. 'nursing provider':ab,ti | 28 |
|  | 1. 'home care':ab,ti | 21,850 |
|  | 1. 'family care':ab,ti | 2,052 |
|  | 1. 'carers':ab,ti | 18,017 |
|  | 1. 'care givers':ab,ti | 3.638 |
|  | 1. 'care giver':ab,ti | 1.366 |
|  | 1. 'spouse caregivers':ab,ti | 340 |
|  | 1. 'caregivers, spouse':ab,ti | 16 |
|  | 1. 'spouse caregiver':ab,ti | 85 |
|  | 1. 'family caregivers':ab,ti | 8.343 |
|  | 1. 'caregiver, family':ab,ti | 143 |
|  | 1. 'caregivers, family':ab,ti | 225 |
|  | 1. 'family caregiver':ab,ti | 1,985 |
|  | 1. {OR 60-78} | 2,894,833 |
|  | 1. 'burden'/exp OR 'pressure'/exp OR 'strain':ab,ti OR 'stress':ab,ti | 1,485,038 |
|  | 1. 79 AND 80 | 74,458 |
|  | 1. 'caregiver burden'/exp | 778,3 |
|  | 1. 81 OR 82 | 81,134 |
|  | 1. 'quality of life'/exp | 491,759 |
|  | 1. 'health-related quality of life':ab,ti | 63,043 |
|  | 1. 'life quality':ab,ti | 13,323 |
|  | 1. 'health related quality of life':ab,ti | 63,043 |
|  | 1. 'hrqol':ab,ti | 26,748 |
|  | 1. {OR 84-88} | 501,999 |
|  | 1. 'length of stay'/exp | 190,350 |
|  | 1. 'stay length':ab,ti | 1,025 |
|  | 1. 'stay lengths':ab,ti | 40 |
|  | 1. 'hospital stay':ab,ti | 127,852 |
|  | 1. 'hospital stays':ab,ti | 12,651 |
|  | 1. 'stay, hospital':ab,ti | 1,477 |
|  | 1. 'stays, hospital':ab,ti | 37 |
|  | 1. 'length of hospital stay':ab,ti | 36,138 |
|  | 1. {OR 90-97} | 283,989 |
|  | 1. 83 OR 89 OR 98 | 846,058 |
|  | 1. 59 AND 99 | 15,625 |
|  | 1. 'randomized controlled trial (topic)'/exp | 183,089 |
|  | 1. 'clinical trials, randomized':ab,ti | 168 |
|  | 1. 'trials, randomized clinical':ab,ti | 17 |
|  | 1. 'controlled clinical trials, randomized':ab,ti | 12 |
|  | 1. 'randomized':ab,ti | 753,934 |
|  | 1. 'randomly':ab,ti | 446,889 |
|  | 1. 'random*':ab,ti | 1,552,152 |
|  | 1. 'placebo':ab,ti | 308,511 |
|  | 1. 'single blind':ab,ti | 18,502 |
|  | 1. 'double blind':ab,ti | 195,917 |
|  | 1. 'triple blind':ab,ti | 928 |
|  | 1. 'randomized controlled trial'/de | 613,233 |
|  | 1. {OR 101-112} | 1,857,053 |
|  | 1. 100 AND 113 | 1,987 |
|  | 1. 100 AND [randomized controlled trial]/lim | 1,018 |
|  | 1. 114 OR 115 | 2,057 |
|  | 1. 'human'/de NOT ('human'/de AND 'animal'/de) | 21,979,548 |
|  | 1. 116 AND 117 | 2,037 |
|  | 1. 118 AND [english]/lim | **1,839** |
| **Cochrane Libiary** |  |  |
|  | 1. MeSH descriptor: [Geriatric Assessment] explode all trees | 1,452 |
|  | 1. MeSH descriptor: [Health Services for the Aged] explode all trees | 441 |
|  | 1. (Assessment, Geriatric):ti,ab,kw | 3,765 |
|  | 1. (Assessments, Geriatric):ti,ab,kw | 484 |
|  | 1. (Geriatric Assessments):ti,ab,kw | 484 |
|  | 1. (Geriatric Health Services):ti,ab,kw | 748 |
|  | 1. (Health Services for the Elderly):ti,ab,kw | 1,068 |
|  | 1. (Health Services, Geriatric):ti,ab,kw | 748 |
|  | 1. (Geriatric Health Service):ti,ab,kw | 400 |
|  | 1. (Health Service, Geriatric):ti,ab,kw | 400 |
|  | 1. (Service, Geriatric Health):ti,ab,kw | 400 |
|  | 1. (Services, Geriatric Health):ti,ab,kw | 748 |
|  | 1. (Health Services for Aged):ti,ab,kw | 8,494 |
|  | 1. (Comprehensive geriatric Assessment):ti,ab,kw | 528 |
|  | 1. (geriatric evaluation and management uni):ti,ab,kw | 0 |
|  | 1. (acute care for elders unit):ti,ab,kw | 17 |
|  | 1. (inpatient geriatric consultation service):ti,ab,kw | 9 |
|  | 1. (mobile acute care of the elderly):ti,ab,kw | 20 |
|  | 1. (home assessment service):ti,ab,kw | 1,204 |
|  | 1. (hospital home assessment service):ti,ab,kw | 572 |
|  | 1. (Outpatient assessment service):ti,ab,kw | 752 |
|  | 1. (Posthospital discharge CGA):ti,ab,kw | 0 |
|  | 1. (Multidimensional Geriatric Assessment):ti,ab,kw | 87 |
|  | 1. (In-home CGA):ti,ab,kw | 6 |
|  | 1. (outpatient CGA):ti,ab,kw | 22 |
|  | 1. (home-based CGA):ti,ab,kw | 4 |
|  | 1. (Hospital-based CGA):ti,ab,kw | 5 |
|  | 1. (Inpatient CGA):ti,ab,kw | 17 |
|  | 1. (Geriatric unit):ti,ab,kw | 478 |
|  | 1. (Specialist geriatric):ti,ab,kw | 141 |
|  | 1. (Acute geriatric):ti,ab,kw | 782 |
|  | 1. (ACE units):ti,ab,kw | 83 |
|  | 1. (GEMU):ti,ab,kw | 8 |
|  | 1. (IGCS):ti,ab,kw | 67 |
|  | 1. (HHAS):ti,ab,kw | 4 |
|  | 1. (OAS):ti,ab,kw | 223 |
|  | 1. {or 1-36} | 14,331 |
|  | 1. (Multidimensional):ti,ab,kw | 2,728 |
|  | 1. (Multidisciplinary):ti,ab,kw | 5910 |
|  | 1. (Multifactorial):ti,ab,kw | 2,705 |
|  | 1. (Interdisciplinary):ti,ab,kw | 2,011 |
|  | 1. {or 38-41} | 12,891 |
|  | 1. (assess*):ti,ab,kw | 494,463 |
|  | 1. (consultation):ti,ab,kw | 10,395 |
|  | 1. (evaluation):ti,ab,kw | 170,572 |
|  | 1. {or 43-45} | 596,698 |
|  | 1. 42 and 46 | 8,001 |
|  | 1. MeSH descriptor: [Geriatrics] explode all trees | 204 |
|  | 1. MeSH descriptor: [Aged] explode all trees | 204,070 |
|  | 1. (elderly):ti,ab,kw | 47,757 |
|  | 1. (old):ti,ab,kw | 53,349 |
|  | 1. (senior):ti,ab,kw | 2,853 |
|  | 1. (Elder):ti,ab,kw | 53,208 |
|  | 1. (geratic):ti,ab,kw | 4 |
|  | 1. (gerontal):ti,ab,kw | 6 |
|  | 1. (older):ti,ab,kw | 53,209 |
|  | 1. {or 48-56} | 317,598 |
|  | 1. 47 and 57 | 2,409 |
|  | 1. 37 or 58 | 16,038 |
|  | 1. MeSH descriptor: [Caregivers] explode all trees | 2,006 |
|  | 1. (Carer):ti,ab,kw | 1,414 |
|  | 1. (long-term care):ti,ab,kw | 21,116 |
|  | 1. (medical care):ti,ab,kw | 45,110 |
|  | 1. (nursing care):ti,ab,kw | 12,743 |
|  | 1. (Care provider):ti,ab,kw | 5,685 |
|  | 1. (Nursing Provider):ti,ab,kw | 392 |
|  | 1. (Family care):ti,ab,kw | 13,464 |
|  | 1. (Home care):ti,ab,kw | 20,342 |
|  | 1. (Primary care provider):ti,ab,kw | 3,173 |
|  | 1. (Carers):ti,ab,kw | 1,971 |
|  | 1. (Care Givers):ti,ab,kw | 311 |
|  | 1. (Care Giver):ti,ab,kw | 192 |
|  | 1. (Spouse Caregivers):ti,ab,kw | 196 |
|  | 1. (Caregiver, Spouse):ti,ab,kw | 196 |
|  | 1. (Caregivers, Spouse):ti,ab,kw | 196 |
|  | 1. (Family Caregivers):ti,ab,kw | 3,123 |
|  | 1. (Caregiver, Family):ti,ab,kw | 2,640 |
|  | 1. (Caregivers, Family):ti,ab,kw | 3,123 |
|  | 1. (Family Caregiver):ti,ab,kw | 2,640 |
|  | 1. {or 60-79} | 93,678 |
|  | 1. (stress):ti,ab,kw | 55,947 |
|  | 1. strain):ti,ab,kw | 7,678 |
|  | 1. (pressure):ti,ab,kw | 142,889 |
|  | 1. (burden):ti,ab,kw | 19,494 |
|  | 1. {or 81-#84} | 213,755 |
|  | 1. 81 and 85 | 18,753 |
|  | 1. MeSH descriptor: [Quality of Life] explode all trees | 23,524 |
|  | 1. (Life Quality):ti,ab,kw | 112,245 |
|  | 1. (Health-Related Quality Of Life):ti,ab,kw | 16,746 |
|  | 1. (Health Related Quality Of Life):ti,ab,kw | 23,528 |
|  | 1. (HRQOL):ti,ab,kw | 5,134 |
|  | 1. {or 88-91} | 112,569 |
|  | 1. MeSH descriptor: [Length of Stay] explode all trees | 7,140 |
|  | 1. (Stay Length):ti,ab,kw | 24,457 |
|  | 1. (Stay Lengths):ti,ab,kw | 576 |
|  | 1. (Hospital Stay):ti,ab,kw | 27,902 |
|  | 1. (Hospital Stays):ti,ab,kw | 1,654 |
|  | 1. (Stay, Hospital):ti,ab,kw | 27,902 |
|  | 1. (Stays, Hospital):ti,ab,kw | 1,654 |
|  | 1. (Length of hospital stay):ti,ab,kw | 17,927 |
|  | 1. {or 93-100} | 35,345 |
|  | 1. 86 or 92 or 101 | 156,549 |
|  | 1. 102 and 59 and 57 in Trials | **3,754** |
