# Supplementary material for: Effectiveness of comprehensive geriatric assessment intervention on quality of life, caregiver burden and length of hospital stay: a systematic review and meta-analysis of randomised controlled trials
Source: BMC Geriatr. 2021 Jun 21;21:377. doi: 10.1186/s12877-021-02319-2 (PMC8218512; doi:10.1186/s12877-021-02319-2)
Supplement: Supplementary file 3 — Additional file 3. Characteristics and summary findings of included studies. [file 12877_2021_2319_MOESM3_ESM.docx]

**Additional file 3** Characteristics and summary findings of included studies

| **Study** | **Intervention model** | **Country** | **Sample size** | **Age,**  **Mean (SD)** | **Intervention professionals** | **Intervention component** | **Control** |
| --- | --- | --- | --- | --- | --- | --- | --- |
| Gayton 1987[28] | CGA-team | Canada | 404  IG: 222  CG: 182 | 78.5  IG: 78.7 (6.5)  CG: 78.3 (6.7) | Geriatrician, geriatric nurse consultant, physiotherapist, occupational therapist, social worker | Comprehensive and coordinated assessment, consultation, suggestion, regular in-hospital follow-up, weekly team meetings, early and coordinated rehabilitation program, discharge planning with emphasis on arranging services and care appropriate to the patients' and families' needs | Usual care |
| Hogan 1987[29] | CGA-team | Canada | 113  IG: 57  CG: 56 | 82.4  IG: 82.2 (6.2)  CG: 83.3 (6.0) | Geriatrician, nurse, physiotherapist | Medical consultation, specific recommendations, daily in-hospital follow-up and check for recommendations implementation, weekly team meetings, emphasis on the management of functional problems as well as discharge planning | Usual care |
| Thomas 1993[30] | CGA-team | United States | 120  IG: 62  CG: 58 | 76.5  IG: 76 (5.4)  CG: 77 (5.4) | Physician, geriatric nurse specialist, home health nurse, medical social worker, dietician, pharmacist, physiotherapist | Individual assessments, formal recommendations through team meeting, in-hospital subsequent visits | Usual care |
| Nikolaus 1999 (CGA+home)[31] | CGA-team and home intervention | Germany | 366  IG: 181  CG: 185 | 81.4 | Nurse, physiotherapist, occupational therapist, social worker, secretary | Comprehensive geriatric assessment, additional in-hospital treatment, post-discharge follow-up treatment, home visit, check for recommendations implementation | Usual care |
| Nikolaus 1999 (CGA)[31] | CGA-team | Germany | 364  IG: 179  CG: 185 | 81.4 | NM | Comprehensive geriatric assessment with recommendations, follow-up usual care at home | Usual care |
| Naglie 2002[32] | CGA-team | Canada | 279  IG: 141  CG: 138 | 84.2  IG: 83.8 (6.9)  CG: 84.6 (7.8) | Geriatrician, senior internal medicine resident, clinical nursing specialist, social worker, physiotherapist, occupational therapist, nurse | Intervention staff working together for a 10-month pilot period before intervention, assessment, daily medical care supervised by an internist-geriatrician, twice-weekly meetings to develop and monitor treatment plans, early mobilization, early participation in self-care and individualized discharge planning, prevention of problems common in elderly patients with hip fracture | Usual care |
| Shyu 2005[33] | CGA-team | China, Taiwang | 137  IG: 68  CG: 69 | 77.7  IG: 77.6 (8.3)  CG: 77.7 (7.1) | Geriatrician, geriatric nurse, physiotherapist, rehabilitation physician | Geriatric assessment/ consultation medical supervision, rehabilitation program(postoperative rehabilitation and plan for hospital discharge), discharge planning service(home visit assessment, environmental modifications, coordinated and monitored the patient’s use of follow-up services) | Usual care |
| Vidan 2005[34] | CGA-team | Spain | 319  IG: 155  CG: 164 | 81.9  IG: 81.1 (7.8)  CG: 82.6 (7.4) | Geriatrician, orthopedic surgeon, nurse, specific social worker, rehabilitation specialist | Geriatric evaluation, weekly interdisciplinary meeting to assess compliance with the plan, comprehensive therapeutic plan, daily visit, responsible for medical care, physical therapy, social support advice, | Usual care |
| Kircher 2007[35] | CGA-team | Germany | 234  IG: 105  CG: 129 | 78.7  IG: 79.0 (6.9)  CG: 78.4 (6.9) | Geriatrician, nurse, social worker, optionally other paramedical staff | Multidimensional assessment, summarising problems and recommendations, at least weekly team meetings, evaluating treatment and implementation of  recommendations, informing family, community services and general practitioner about recommendations follow-up call, necessary telephone consultation | Usual care |
| Pitkala 2008[36] | CGA-team | Finland | 174  IG: 87  CG: 87 | 83.5  IG: 84 (5.6)  CG: 83 (6.2) | Geriatrician, social worker, occupational therapist, physiotherapist, nurse | Comprehensive geriatric assessment and treatment, avoiding conventional neuroleptics, physiotherapy, general geriatric interventions, comprehensive discharge planning(consultation of social worker, occupational therapists home visit, discharge planning with the caregivers) | Usual care |
| Prestmo 2015[37] | CGA-team | Norway | 397  IG: 198  CG: 199 | 83.3  IG: 83.4 (5.4)  CG: 83.2 (6.4) | Geriatrician, registered nurse, licensed practical nurse, physiotherapists, occupational therapist | Structured, systematic interdisciplinary comprehensive geriatric assessment and care, early mobilisation and initiation of rehabilitation, early discharge planning | Usual care |
| Partridge 2017*[38] | CGA-team | United Kingdom | 209  IG: 104  CG: 105 | 75.5  IG: 75.5 (6.6)  CG: 75.5 (6.3) | Geriatrician, clinical nurse specialist, social worker, occupational therapist | Comprehensive geriatric assessment and optimization, individualized care plan（advice regarding the prevention and management of anticipated postoperative complications） | Usual care |
| Applegate 1990*[39] | CGA-unit | United States | 155  IG: 78  CG: 77 | 78.8  IG: 79.4 (7.0)  CG: 78.1 (7.6) | Physician, rehabilitation nurse, social worker, physiotherapist, occupational therapist, psychologists, social workers, nutritionists, specialists in speech therapy and audiology | Interdisciplinary assessment, weekly team meetings, medical treatment and rehabilitation care with weekly progress evaluation when needed | Usual care |
| Karppi 1995[40] | CGA-unit | Finland | 312  IG: 104  CG: 208 | 78.3  IG: 78.5 (4.3)  CG: 78.2 (4.7) | Doctor, nurse, auxiliary nurses, assistants, secretary, psychologist, occupational therapist, physiotherapists, part-time social worker, consulting specialists when needed. | Rehabilitation plan, medication changes, specialist consultations, psychiatrist visiting the ward regularly once a week | Usual care |
| Covinsky 1997[41] | CGA-unit | United States | 650  IG: 326  CG: 324 | 80  IG: 80 (7)  CG: 80 (7) | Nursing and medical director, social worker, psychologist, occupational therapist, dietitian, primary nurse, house staff, attending physician | Specially designed environment, patient-centred care, planning for discharge, review of medical care, prevention of disability, specific needs daily assessment | Usual care |
| Asplund 2000[42] | CGA-unit | Sweden | 413  IG: 190  CG: 223 | 80.95  IG: 80.9 (95％CI: 80.1-81.9)  CG: 81.0 (95％CI: 80.3-81.8) | Geriatrician, internist, nurse, psychologist, dietitian, occupational therapist | One-week education period for the staff with emphasis on the principles of interdisciplinary and geriatric working forms and on ethical issue, 3-week run-in period of the AGW before study; optimize the conditions for treatment, nursing, daily medical team meetings, early assessment, start of rehabilitation, interdisciplinary team work, planning of discharge | Usual care |
| Counsell 2000[43] | CGA-unit | United States | 1531  IG: 767  CG: 764 | 79.5  IG: 80 (7)  CG: 79 (7) | Geriatrician medical director, attending and resident physician, geriatric clinical nurse specialist, social worker, physical therapist | Specially designed environment, daily interdisciplinary team meetings, patient-centred care, including nursing care plans for prevention of disability and rehabilitation; planning for patient discharge to home; review of medical care to prevent iatrogenic illness | Usual care |
| Cohen 2002(unit)[44] | CGA-unit | United States | 1388  IG: 692  CG: 696 | 74.2 | Senior geriatrician, specialist nurse, social worker, physiotherapist,  occupational therapist, dieticians, pharmacist | Specific instructions to complete the history taking and physical examination, comprehensive assessment, developing a list of problems, plan of care, team meetings at least twice a week, preventive and management services | Usual care |
| Saltvedt 2004[45] | CGA-unit | Norway | 254  IG: 127  CG: 127 | 82  IG: 82 (5)  CG: 82 (5) | Geriatrician, resident, nurse, occupational therapist, physical therapist | Interdisciplinary and comprehensive assessment, regular interdisciplinary meetings to set goals, early mobilization/rehabilitation, prevention of complications, early discharge planning, home services aids when needed | Usual care |
| Ekerstad 2016[46] | CGA-unit | Sweden | 408  IG: 206  CG: 202 | 85.7  IG: 85.7 (5.3)  CG: 85.6 (5.6) | Specialist in internal medicine, family medicine and/or geriatrics, occupational therapists, physiotherapists, specialized admission and discharge nurses | Systematic structured interdisciplinary CGA and care: somatic and mental health, medication review, functional and activity ability including early rehabilitation, social situation, early discharge planning | Usual care |
| Silverman 1995[47] | OAS | United States | 442  IG: 239  CG: 203 | 74.6  IG: 74.6 (7.46)  CG: 74.6 (7.35) | Geriatrician, geriatric nurse, geriatric social worker | Comprehensive outpatient evaluation, comprehensive treatment plan, family conference to discuss findings and the treatment plan, discussing and providing recommendations on addressing issues including caregivers’ degree of stress and the effects of the intervention on them | Usual care |
| Reuben 1999[48] | OAS and adherence intervention | United States | 363  IG: 180  CG: 183 | 75.9  IG: 75.8 (6.1)  CG: 75.9 (5.7) | Social worker, gerontological nurse practitioner/geriatrician team, physical therapist | In-depth standardized, comprehensive geriatric assessment, interdisciplinary team meeting, adherence intervention aimed both at patients and their physicians(conveying CGA recommendations to physician in details, review the team’s recommendations, ensure patients understand and agree recommendations, interact proactively with physicians) | Usual care |
| Burns 2000[49] | OAS | United States | 98  IG: 49  CG: 49 | 71.3  IG: 71.7 (6.3)  CG: 70.8 (3.7) | Physician, nurse practitioner, social worker or clinical psychologist | Initial comprehensive assessment, continuing long-term management, team members developing a cohesive, cooperative approach to patient care delivery | Usual care |
| Weuve 2000[50] | OAS | United States | 368  IG: 294  CG: 274 | 79.6 | Geriatrician, nurse, social worker, gerontological nurse practitioner | First home visit for comprehensive assessment, integrated set of treatment goals and plans, subsequent visits to provide medical treatment, care management, educational information, consultation, assistance with advance directives, referral to agencies and other professionals as needed, counselling caregivers and referring them to other providers and resources | Usual care |
| Cohen 2002(outpatient)[44] | OAS | United States | 1388  IG: 694  CG: 694 | 74.2 | Senior geriatrician, specialist nurse, social worker, physiotherapist, occupational therapist, dieticians, pharmacist | Specific instructions to complete the history taking and physical examination, comprehensive assessment, developing a list of problems, plan of care, team meetings at least twice a week, preventive and management services | Usual care |
| Ekdahl 2015[51] | OAS | Sweden | 382  IG: 208  CG: 174 | 82.5  IG:82.3 (4.6)  CG:82.7 (5.1) | Nurse, geriatrician/ resident physician, care manager, occupational therapist, physiotherapist, dietician, administrative assistant. other professionals (e g, dental hygienist, psychologist) | Comprehensive geriatric assessment, individually tailored care, team meetings, reassessment, preventive intervention activities, follow-up visits(telephone or ambulatory or home visits), informing patients of available forms of support, ensuring patients understand new prescriptions, advice and counsel, providing further information when admitting to hospital | Usual care |
| Zintchouk 2018*[52] | OAS | Denmark | 368  IG: 185  CG: 183 | 78.6  IG: 78.8 (8.3)  CG: 78.4 (7.9) | Geriatrician, nurse, physical therapist, occupational therapist, nutritionist, assistant nurse | Assessment by the geriatrician, defining actual problems, expectations and aims, individual disease management and coping, focus on the potentially functional deterioration, medication adjustment, interventional follow up by a geriatrician | Usual care |
| Counsell 2007[53] | HAS | India | 951  IG: 474  CG: 477 | 71.8  IG: 71.8 (5.6)  CG: 71.8 (5.6) | Advanced practice nurse, mental health social worker, geriatrician, primary care physician, pharmacist, physical therapist, community-based services liaison | Comprehensive geriatric assessment, weekly team meeting to develop an individualized care plan, discussing and modifying the plan with the primary care physician, implementing the plan consistent with the patient’s goals, ≥1 follow-up visit, 1 telephone or face-to face contact per month, a face-to face home visit after any emergency department visit or hospitalization, annual in-home reassessment, | Usual care |
| Fairhall 2015[54] | HAS | Australia | 241  IG: 120  CG: 121 | 83.3  IG: 83.4 (5.81)  CG: 83.2 (5.91) | Physiotherapists, geriatrician, rehabilitation physician, dietician, nurse | Detailed assessment, interventions based on frailty characteristics, medical management, regular interdisciplinary case-meetings and case management, ongoing reassessment, home visits, | Usual care |
| Edmans 2013[55] | HHAS | United Kingdom | 433  IG: 216  CG: 217 | 82.9  IG: 83.1 (6.7)  CG:82.8 (7.0) | Geriatrician, general practitioner, nurse, physiotherapist, occupational therapist | Assessment before discharge to coordinate the delivery of necessary additional immediate care or aftercare(review of diagnoses; drug review; further assessment; advance care planning; liaison with primary care, intermediate care, and specialist community services), follow-up home visits, phone calls, clinic visits | Usual care |

CGA, comprehensive geriatric assessment; OAS, Outpatient Assessment Service; HHAS, Hospital Home Assessment Service; HAS, Home Assessment Service; IG, intervention group (comprehensive geriatric assessment intervention group); CG, control group (usual care group); CI, confidence interval; NM, not mentioned.

^*^ study not included in meta-analysis.
