# Supplementary figures and images for: Effectiveness of comprehensive geriatric assessment intervention on quality of life, caregiver burden and length of hospital stay: a systematic review and meta-analysis of randomised controlled trials
Source: BMC Geriatr. 2021 Jun 21;21:377. doi: 10.1186/s12877-021-02319-2 (PMC8218512; doi:10.1186/s12877-021-02319-2)

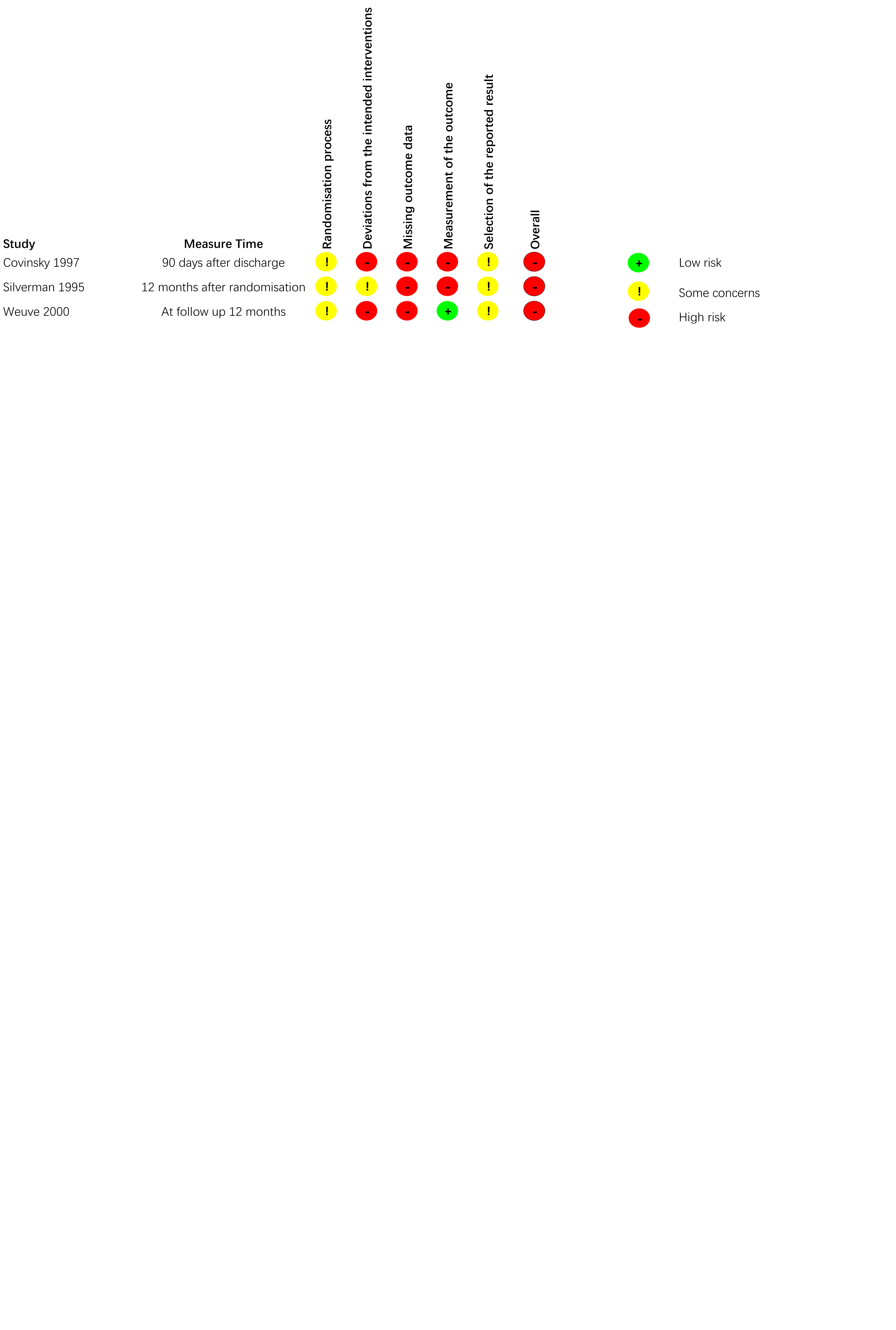

Supplement: Supplementary file 4 — Additional file 4. Risk of bias of included studies about the outcome indicator caregiver burden. [file 12877_2021_2319_MOESM4_ESM.tif]

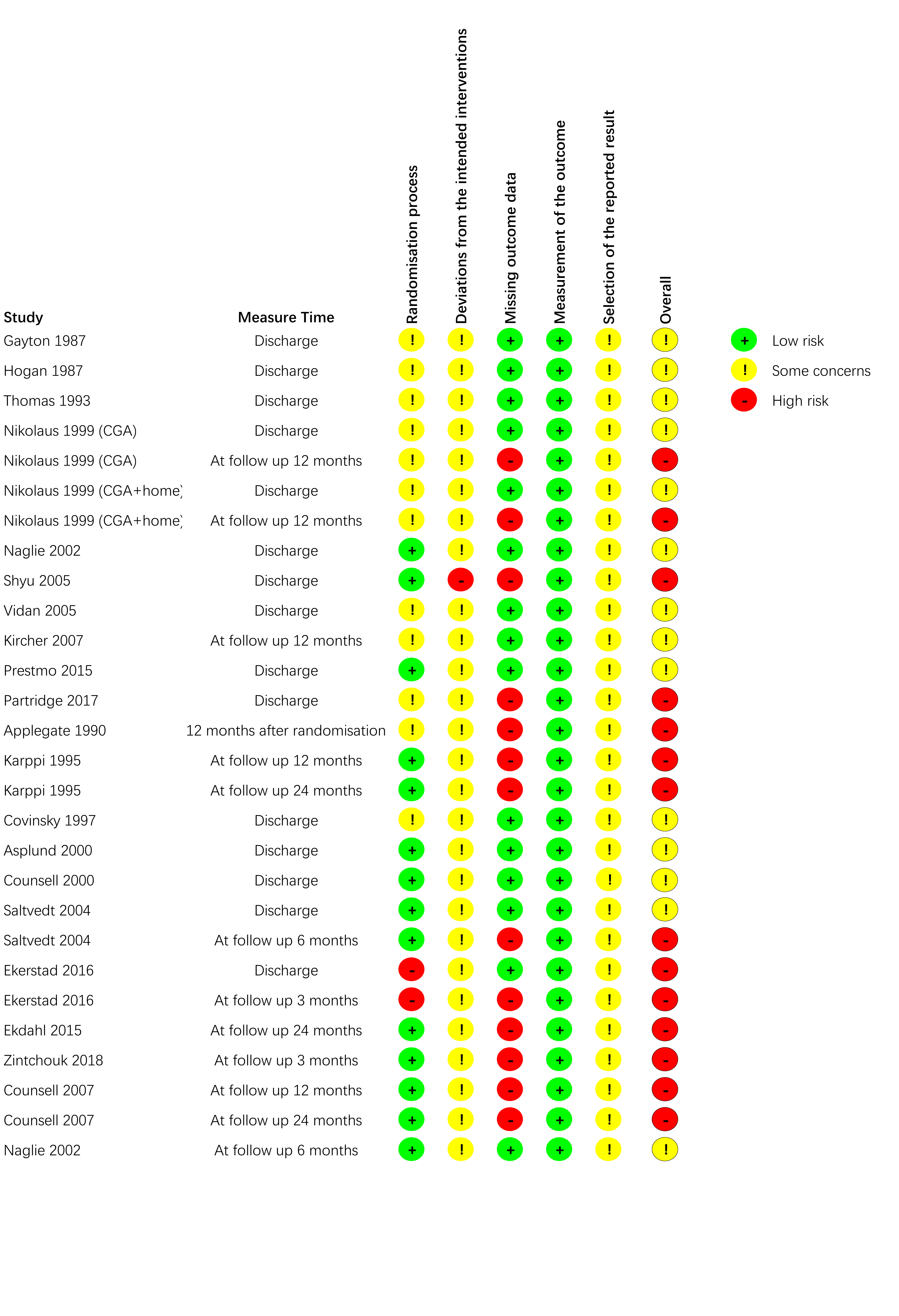

Supplement: Supplementary file 5 — Additional file 5. Risk of bias of included studies about the outcome indicator length of hospital stay. [file 12877_2021_2319_MOESM5_ESM.tif]

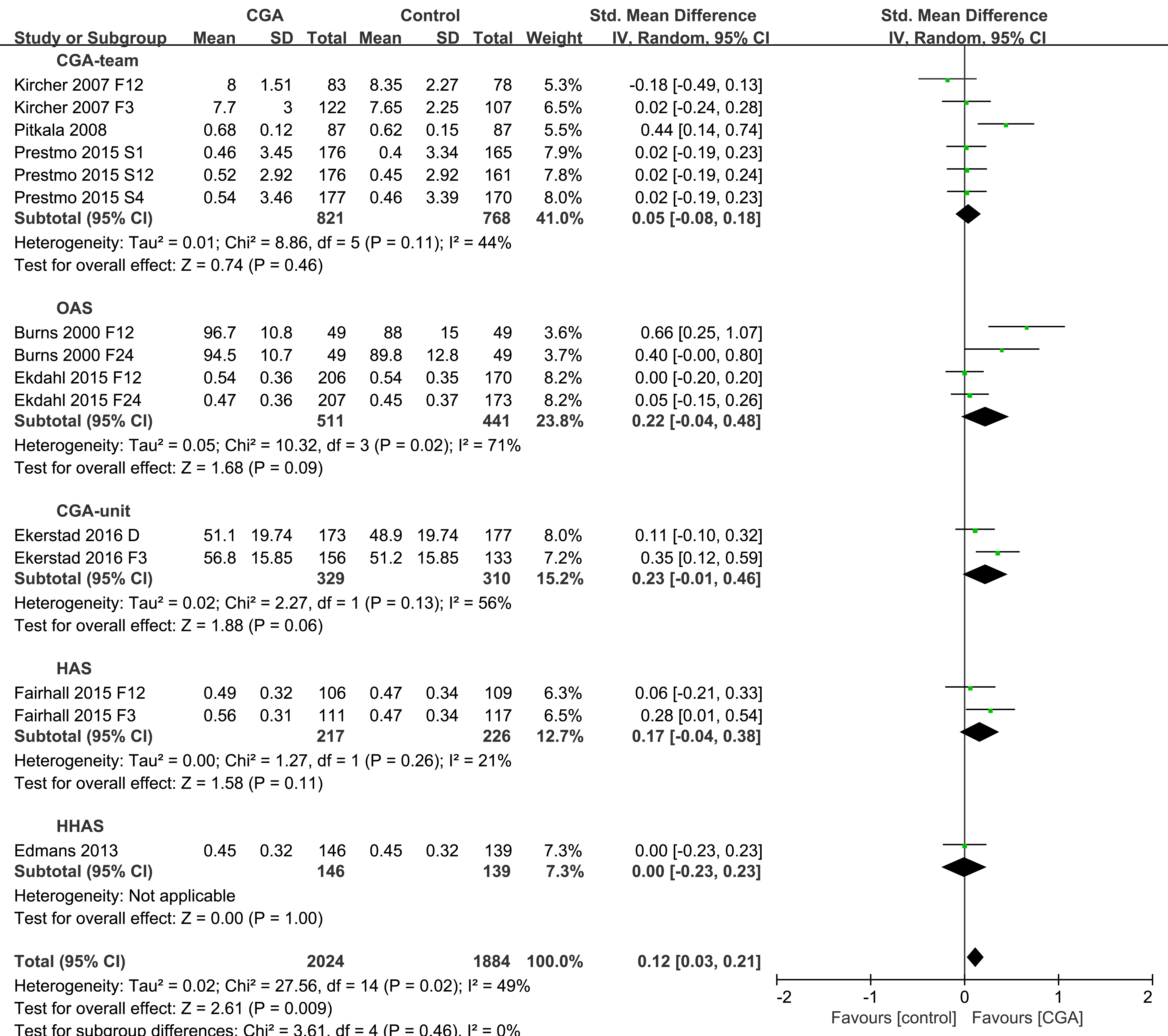

Supplement: Supplementary file 9 — Additional file 9. Quality of life-subgroup analysis based on different intervention models. [file 12877_2021_2319_MOESM9_ESM.tif]

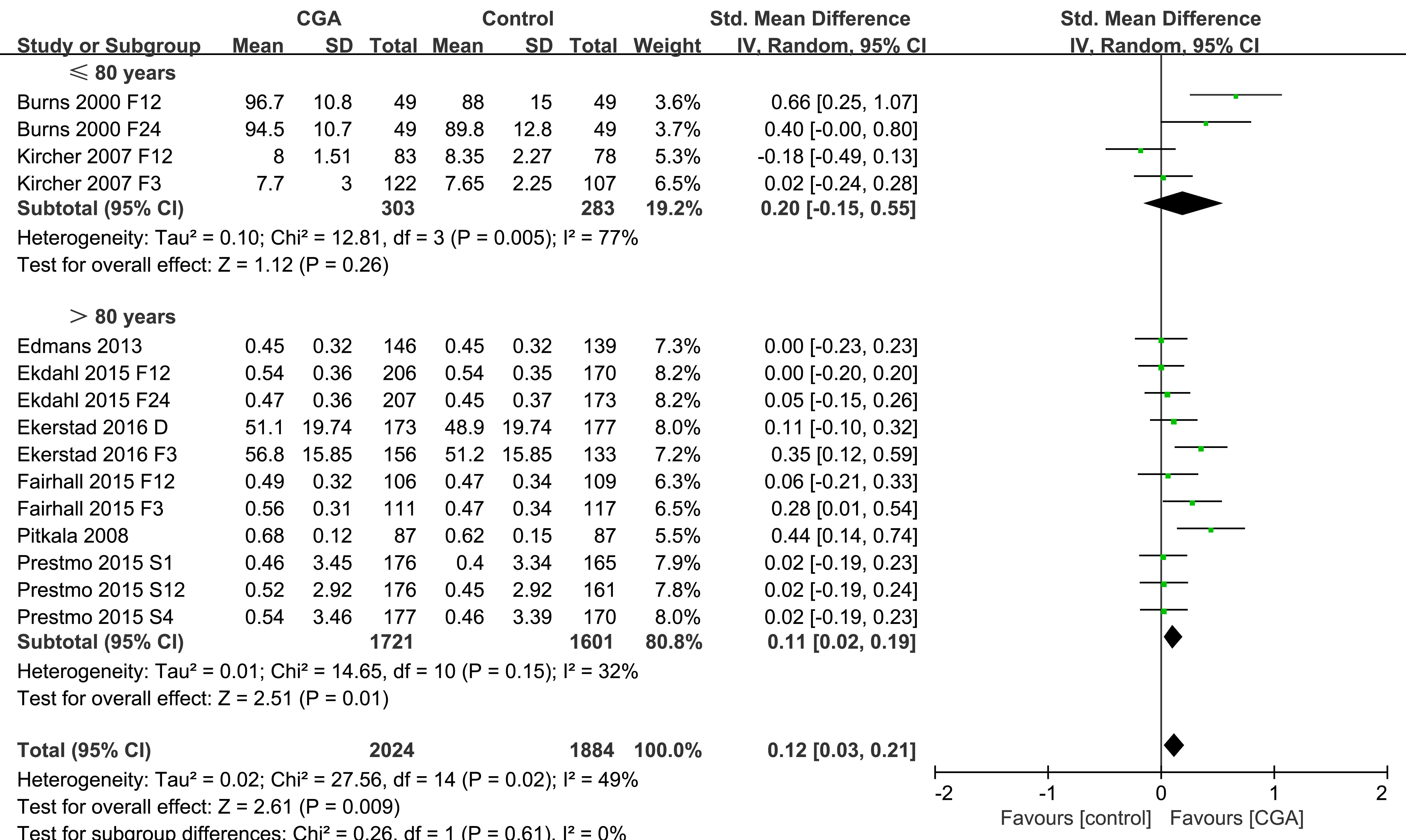

Supplement: Supplementary file 10 — Additional file 10. Quality of life-subgroup analysis based on participants' age. [file 12877_2021_2319_MOESM10_ESM.tif]

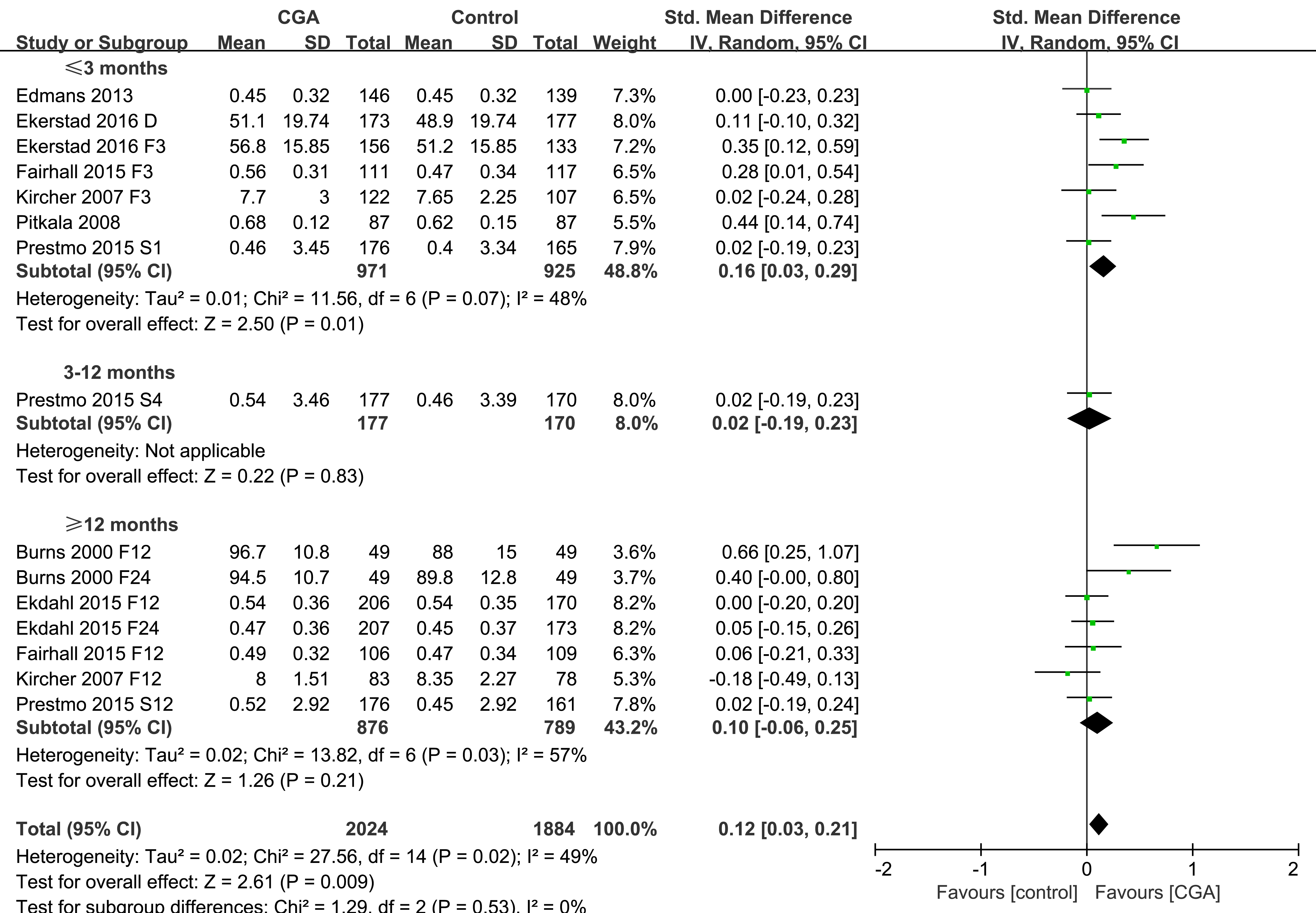

Supplement: Supplementary file 11 — Additional file 11. Quality of life-subgroup analysis based on outcome measure time. [file 12877_2021_2319_MOESM11_ESM.tif]

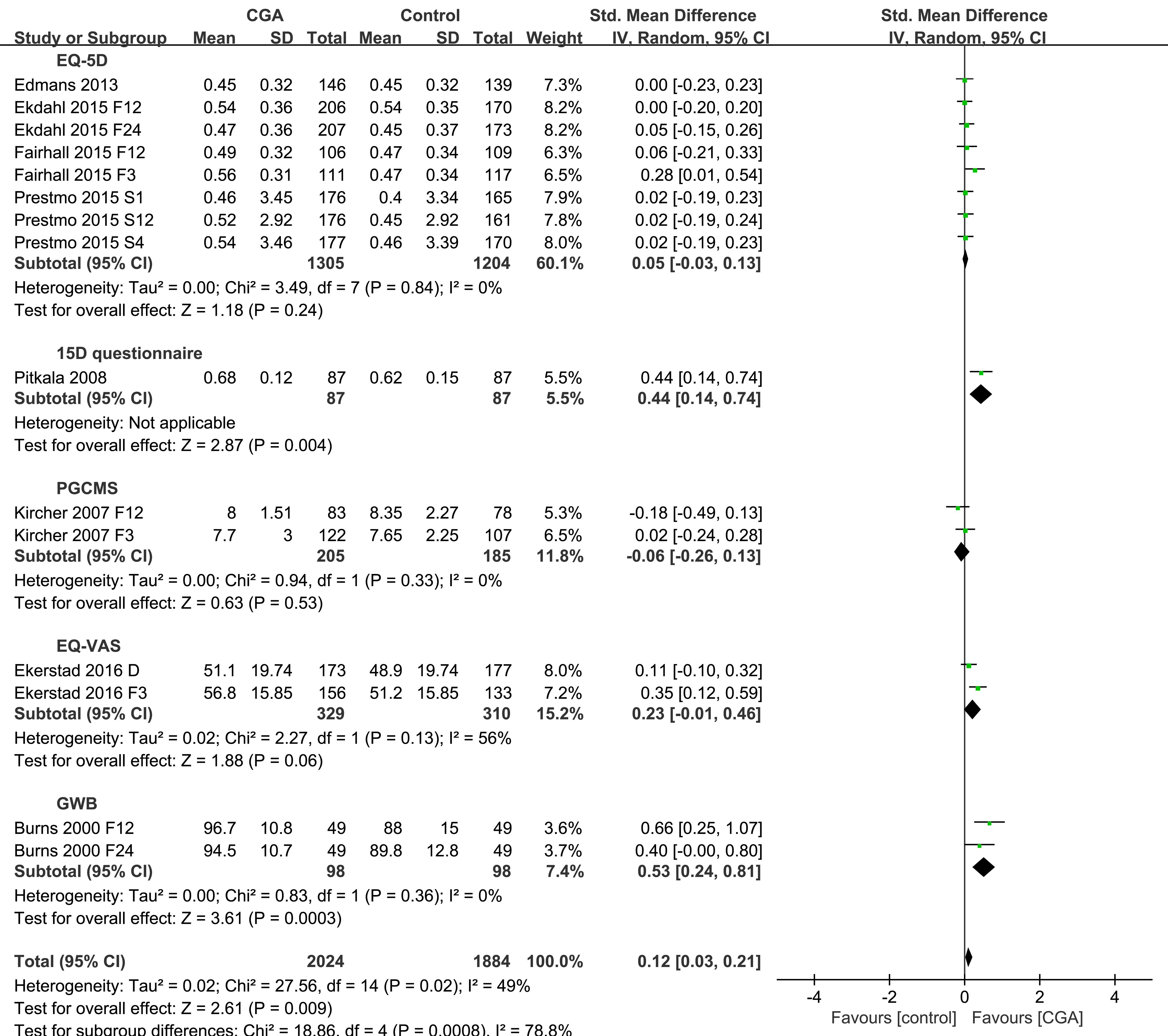

Supplement: Supplementary file 12 — Additional file 12. Quality of life-subgroup analysis based on evaluation instrument. [file 12877_2021_2319_MOESM12_ESM.tif]
