# Supplementary material for: Effectiveness of comprehensive geriatric assessment intervention on quality of life, caregiver burden and length of hospital stay: a systematic review and meta-analysis of randomised controlled trials
Source: BMC Geriatr. 2021 Jun 21;21:377. doi: 10.1186/s12877-021-02319-2 (PMC8218512; doi:10.1186/s12877-021-02319-2)
Supplement: Supplementary file 8 — Additional file 8. Sensitivity analyses for primary outcome. [file 12877_2021_2319_MOESM8_ESM.docx]

**Additional file 8** Sensitivity analyses for primary outcome

| **Pooled analysis** | **Study number**  **(analyses number)** | **Effect**  **SMD/MD (95% CI)** | **P value** | **Heterogeneity**  **I^2^ (%)** |
| --- | --- | --- | --- | --- |
| **Quality of life level** |  |  |  |  |
| All groups | 8(15) | 0.12 [0.03, 0.1] | 0.009 | 49 |
| including studies with low risk of measurement of the outcome | 4(7) | 0.04 [-0.05, 0.13] | 0.41 | 0 |
| **Length of hospital stay** |  |  |  |  |
| All groups | 17(22) | -1.04 [-3.57, 1.49] | 0.42 | 100 |
| including studies with some concerns in risk of bias | 13(13) | 0.15 [-2.80, 3.10] | 0.92 | 100 |
